# Updating the bionomy and geographical distribution of *Anopheles* (*Nyssorhynchus*) *albitarsis* F: A vector of malaria parasites in northern South America

**DOI:** 10.1371/journal.pone.0253230

**Published:** 2021-06-17

**Authors:** Miguel A. Zúñiga, Yasmin Rubio-Palis, Helena Brochero

**Affiliations:** 1 Escuela de Microbiología, Facultad de Ciencias, Departamento Francisco Morazán, Universidad Nacional Autónoma de Honduras, Tegucigalpa, Honduras; 2 Departamento Clínico Integral, Facultad de Ciencias de la Salud, sede Aragua, Universidad de Carabobo, Maracay, Estado Aragua, Venezuela; 3 Centro de Estudios de Enfermedades Endémicas y Salud Ambiental (CEEESA), Servicio Autónomo Instituto de Altos Estudios “Dr. Arnoldo Gabaldon”, Maracay, Estado Aragua, Venezuela; 4 Departamento de Agronomía, Facultad de Ciencias Agrarias, Bogotá, Universidad Nacional de Colombia, Bogotá, Distrito Capital, Colombia; Universitat Wien, AUSTRIA

## Abstract

*Anopheles albitarsis* F is a putative species belonging to the Albitarsis Complex, recognized by rDNA, mtDNA, partial *white* gene, and microsatellites sequences. It has been reported from the island of Trinidad, Venezuela and Colombia, and incriminated as a vector of malaria parasites in the latter. This study examined *mitochondrially encoded cytochrome c oxidase I* (*MT-CO1*) sequences of *An*. *albitarsis* F from malaria-endemic areas in Colombia and Venezuela to understand its relations with other members of the Complex, revised and update the geographical distribution and bionomics of *An*. *albitarsis* F and explore hypotheses to explain its phylogenetic relationships and geographical expansion. Forty-five *MT-CO1* sequences obtained in this study were analyzed to estimate genetic diversity and possible evolutionary relationships. Sequences generated 37 haplotypes clustered in a group where the genetic divergence of Venezuelan populations did not exceed 1.6% with respect to Colombian samples. *Anopheles albitarsis* F (π = 0.013) represented the most recent cluster located closer to *An*. *albitarsis* I (π = 0.009). Barcode gap was detected according to Albitarsis Complex lineages previously reported (threshold 0.014–0.021). *Anopheles albitarsis* F has a wide distribution in northern South America and might play an important role in the transmission dynamics of malaria due to its high expansion capacity. Future studies are required to establish the southern distribution of *An*. *albitarsis* F in Venezuela, and its occurrence in Guyana and Ecuador.

## Introduction

For over 40 years, the *Anopheles* (*Nyssorhynchus) albitarsis* complex has been considered a species complex based on chromosomal inversions [1, 2], allozymes [3, 4], random amplified polymorphic DNA-polymerase chain reaction (RAPD-PCR) [3, 5, 6] and, morphology and behavior [7, 8]. At present, the Albitarsis Complex includes five formally described species: *An*. *albitarsis* Lynch-Arribálzaga sensu stricto, *An*. *deaneorum* Rosa-Freitas, *An*. *marajoara* Galvão & Damasceno, *An*. *oryzalimnetes* Wilkerson & Motoki and *An*. *janconnae* Wilkerson & Sallum, and five putative species based on molecular studies: *An*. *albitarsis* F [9], *An*. *albitarsis* G [10, 11], *An*. *albitarsis* I [11, 12], *An*. *albitarsis* H [11] and *An*. *albitarsis* J [13]. Geographical distribution restriction has been found for some of these species [11, 14].

*Anopheles albitarsis* F was reported for the first time from Puerto Carreño, Vichada, Colombia [9] based on *white* gene, rDNA-ITS2 and consistently supported by microsatellite sequences [15, 16] and DNA *mitochondrially encoded cytochrome c oxidase I* (*MT-CO1*) sequences [11, 13], although morphologic diagnostic characters ranges for natural adult female populations have been reported [17, 18]. This species has been reported from coastal, piedmont, savannah, and interior lowland forest ecoregions [19, 20] in Colombia, the island of Trinidad and Venezuela [9, 11, 17, 21]. Natural populations of *An*. *albitarsis* F (as *An*. *marajoara*) from Colombia have been found naturally infected with *Plasmodium falciparum* and *P*. *vivax* [21, 22]. *Anopheles albitarsis* I, its sister species, occurs in northern Colombia and in sympatry with *An*. *albitarsis* F in Norte de Santander, Colombia and Zulia, north western Venezuela, near the border with Colombia [11, 12, 16, 23, 24]. It is considered that previous reports on geographic distribution, bionomy, and malaria vector incrimination of *An*. *albitarsis* sensu lato, *An*. *marajoara* and, *An*. *allopha* (*nomen dubium*) collected in Colombia, Trinidad and Tobago and Venezuela corresponded to either of these two lineages.

In Colombia, two genetic pools for these denominations were characterized based on microsatellite sequences [15, 16], one recognized now as *An*. *albitarsis* F, distributed east of the Eastern Andean Cordillera that corresponds to the cis-Andean genetic pool [9, 11, 21], and another, now recognized as *An*. *albitarsis* I [11, 24] corresponding to the trans-Andean genetic pool, distributed along the valleys between the Central and Eastern Andean Cordillera and lowlands of northern Colombia. Notably, Brochero et al. [16] found a higher number of hybrids between the cis and trans-gene pools as they were not completely isolated. It means that some foreign mosquitoes in a given population could be constituted of migrants with no genetic interchange with the original mosquitoes. The migration of individuals and the gene-flow levels detected were elevated, despite the fact that an important geographic barrier such as the Eastern Andean Cordillera separated the populations studied providing only partial differentiation of the two gene pools but addressing it. These genetic pools showed that their populations were remarkably similar, with divergence times of approximately 29 years when a mutation rate of 6.3x10^-6^ and twelve generations per year were estimated. These populations were characterized for having high historical effective numbers with a tendency of expansion in the absence of bottleneck events [15].

Rubio-Palis et al. [25], concluded that *An*. *marajoara* was the only species from the Albitarsis Complex present in Venezuela, based on morphometric analysis of 12 populations along the range of distribution of *An*. *albitarsis* s. l. in this country, and RAPD-PCR of one population from the center of the country. Based on the barcode region of mtDNA, sequences from Zulia and Cojedes States in western Venezuela and, a sequence from Portuguesa State, Venezuelan Llanos, assumed by Lehr et al. [26] to be *An*. *janconnae* (GenBank: DQ076234), were later confirmed as *An*. *albitarsis* F by Ruíz-López et al. [11]. Shortly after, Rubio-Palis et al. [17] reported *An*. *albitarsis* F for the first time from a malaria endemic area in Sucre Municipality, Bolívar State, south of the Orinoco River, which suggested a much wider range of distribution for this taxon in Venezuela. Additionally, *An*. *albitarsis* F has been confirmed from the island of Trinidad [11, 13] where previously Chadee and Wilkerson [27] had identified *An*. *marajoara* based on RAPD-PCR and ITS2-rDNA.

In the present study, we report an analysis of *MT-CO1* sequences of *An*. *albitarsis* F samples collected in malaria-endemic areas of Colombia and Venezuela, a review of its bionomy and hypotheses of its origin and current geographical distribution.

## Materials and methods

### Study areas and mosquito collections

Specimens from Venezuela were collected in two localities: Calabozo, Guárico State (8°58’ N, 67°25’ W) in March 1999, preserved in 96% isopropanol, and San Rafael, Bolívar State (06° 47 N, 61 °34 W) in March 2014, preserved dry in silica gel. Collections were conducted by researchers and public health inspectors from the Ministry of Health involved in the Malaria Control Program who therefore did not need specific ethical clearance and no permits were required; permission for collecting in private properties was received from owners prior to collections. Calabozo is the capital of Francisco de Miranda Municipality; this area is characterized by large rice field plantations. Malaria was eradicated there in the early 1950’s and the incriminated vector was *An*. (*Nys*.) *darlingi* Root [28]. At present, malaria cases have been reported from the area, but no entomological information is available. San Rafael is a gold mining camp located in Sifontes Municipality, which in 2018 reported a total of 150,115 malaria cases [29]; incriminated vectors were *An*. *darlingi*, *An*. *albitarsis* s.l. (as *An*. *marajoara*) and *An*. (*Anopheles*) *neomaculipalpus* Curry based on their sporozoite and malaria entomological inoculation rates [30, 31]. Official records of malaria cases are usually sub-estimated due to self-medication, illegal migration from neighboring countries, and the limited diagnosis and treatment activities due to the economic crisis [32].

Specimens from Colombia were collected in three endemic malaria areas of the Departments of Meta, Guaviare and Vichada. Samples from Meta were collected in Puerto Gaitán (04°19′N, 72°05′W), where an urban *P*. *falciparum* malaria outbreak was reported during sampling [33]; specimens from Vichada were collected in peri urban areas of Puerto Carreño (06°11′16″N, 67°28′23″W), the capital of Vichada Department near the border with Venezuela; and, samples from Guaviare (02°34′43.8″N, 72°37′31.6″W) corresponded to peri urban localities where malaria transmission was enhanced by proliferation and intensification of the cultivation of illicit crops, internal armed conflict, intensive land use and immigration of people from hyperendemic malaria areas [34]. Detailed description of study sites and collection methods have been previously published [21, 34, 35]. Taxonomic identification was based on morphological characters [36–38] and DNA *MT-CO1* barcode sequences available in the GenBank and the Bold System public databases. Colombian samples were collected based on the protocols of the Malaria Vector Biology in Brazil project funded by The National Institute of Health (NIH, R01 AI5413), U.S., and the Universidad Nacional de Colombia, Quipu Code 201010012197. Collection of wild adult mosquitoes by human landing catches was conducted under an informed consent agreement using a protocol and collection procedures that were reviewed and approved by the Ethics Committee of the Faculty of Medicine of the Universidad Nacional de Colombia and by the Institutional Review Board of the New York State Department of Health, protocol No. 02–028. No additional permits were necessary.

### DNA extraction and DNA *MT-CO1* analysis

Total genomic DNA was extracted from three Venezuelan specimens, randomly chosen from each group of mosquitoes collected in each geographical locality: one from Calabozo and two from San Rafael. Additionally, *g*DNA of 17 mosquitos from Guaviare, 5 from Meta and 20 from Vichada, Colombia were obtained. DNA extractions were done by using the DNeasy blood and tissue kit (QIAGEN^®^, Hilden, Germany) but DNA was eluted in 30 μL of buffer AE. The DNA *MT-CO1* gene sequence (corresponding to the DNA barcode region) was amplified using the universal primers [39] and PCR conditions corresponded to those described by Ruíz-López et al. [11]. All PCR reactions included negative controls. PCR products were subjected to bidirectional sequencing by Corporación Corpogen (Bogotá, Colombia, www.corpogen.org). Forward and reverse sequences were manually edited based on the chromatograms and consensus sequences were obtained by alignment using ClustalW [40] algorithm under Geneious v7.1.7 software (https://www.geneious.com). The *MT-CO1* data sets amino acids translation did not show stop codons, meaning no pseudogenes were included in the analyses. Saturation levels between transition and transversion rates in relation to genetic distances were checked using the DAMBE v7.0 [41]. Sequence identification of species was confirmed by comparison to publicly available sequence data in the GenBank using the BLASTn algorithm [42] and using the BOLD Identification System (IDS) of the BOLD Systems database [43]. The intra and inter-specific genetic distances were calculated in MEGA v10.0 software [44] based on the Kimura-2-parameter (K2P) model [45].

### Phylogenetic analyses based on barcode *MT-CO1* sequences

Two analyses were done to estimate phylogenetic relationships. First, we analysed 45 sequences of *An*. *albitarsis* F obtained in this study and 123 publicly available sequences representative of the Albitarsis Complex members located in northern South America (N = 57 *An*. *albitarsis* F; N = 50 *An*. *albitarsis* I; N = 11 *An*. *marajoara* and N = 5 *An*. *janconnae*). Second, the analysis was done with 12 sequences from this study (N = 3 Venezuela; N = 9 Colombia) and 88 publicly available sequences representing all members of the Albitarsis Complex (N = 7 *An*. *albitarsis* s.s.; N = 9 *An*. *oryzalimnetes*; N = 11 *An*. *marajoara*; N = 5 *An*. *deaneorum*; N = 5 *An*. *janconnae*; N = 24 *An*. *albitarsis* F; N = 8 *An*. *albitarsis* G; N = 5 *An*. *albitarsis* H and, N = 14 for *An*. *albitarsis* I). The phylogenetic relationships were inferred using Neighbor Joining (NJ) and Bayesian inference (BI) methods. The NJ trees were constructed using the K2P distances model: branch support was assessed by bootstrapping with 1,000 replicates (Bootstrap values-BSV) [46, 47] using MEGA v10.0 software [44]. Bayesian inference (BI) analyses were performed in BEAST v1.10.4 [48] using the nucleotide substitution model HKY+I+G [49] previously selected with the Akaike Information Criterion (AIC) in the jModelTest2 program [50]. The Markov chain Monte Carlo (MCMC) algorithm was executed in duplicate runs of 10 million states each, sampling every 10,000 steps. The convergence of the MCMC chains was checked for effective sample size (ESS) values greater than 200 for each estimated parameter using Tracer v.1.7.1 [51]. The maximum credibility tree was determined using TreeAnnotator v1.10.4 after 25% burn-in and visualized with FigTree v1.4.3 [52]. Bayesian posterior probabilities (BPP) were used to assess nodal support. *Anopheles (Nys*.*) braziliensis* Chagas (GenBank: DQ076236) and *An*. *darlingi* (GenBank: DQ076238) were used as outgroup taxa.

Haplotypes were determined using DnaSP v6.12 [53]. Genetic diversity within populations was estimated by computing haplotype diversity (Hd) [47] and nucleotide diversity (*π*) [54]. Genealogical relationships were analysed through haplotype networks using PopART v1.7 [55] based on the TCS algorithm [56] to show relationships among the individuals sampled from different locations.

### Analysis of published records of geographic distribution and bionomics of *Anopheles albitarsis* s.l., north of the Amazon River

Based on the revisions by Linthicum [36] and Sinka et al. [57] on the geographic distribution and bionomics of *An*. *albitarsis* s.l., a review protocol was established and agreed upon by all authors. The Guidelines from the Cochrane Handbook for Systematic Review was followed as standard methodology [58]. Online scientific bibliographic databases PubMed, LILACS, Web of Science and Scielo were searched using *Anopheles albitarsis*, *An*. *marajoara* and *An*. *allopha* as key words search terms followed by the Boolean operator “and” combined with one of each of the following ‘free text’ terms in succession: ‘entomological surveillance’, ‘larval collection’, ‘adult collection’, ‘resting collection’, ‘landing collection’, ‘vector density’, ‘geographical distribution’. The reference list of each of the included studies was also searched, and “grey literature” was sought by communication with authors for cited unpublished documents. The resulting citation library was then reviewed and refined, retaining all references that met one or more of the following criteria for inclusion: 1) The reported study was conducted in either of the following countries: Costa Rica, Panamá, Colombia, Venezuela, Trinidad and Tobago, Guyana, Suriname, French Guiana, and the Brazilian States of Amazonas, Roraima, Pará and Amapá, located north of the Amazon River; 2) the survey provided specific information on location to a precision of administrative unit level (municipality) or higher; 3) the surveys provided species-level information; 4) the surveys provided data on bionomics; 5) the surveys provided data on malaria vector incrimination. No limits were placed on year of publication and language. Globally, the literature search resulted in 1,837 publications or reports containing potential data to be reviewed. Of these publications, 621 were selected for full evaluation, of which 112 fulfilled the inclusion criteria.

The geographic distribution for *Anopheles albitarsis* s.l., *An*. *albitarsis* F, *An*. *albitarsis* I, *An*. *marajoara* and *An*. *janconnae* were plotted on a map using QGIS version 3.4 [59] based on images from Natural Earth raster (public domain: https://www.naturalearthdata.com) and data from published records extracted from the literature reviewed in this study.

## Results

### DNA extraction and DNA *MT-CO1* analysis of *Anopheles albitarsis* F

Forty-five *MT-CO1* sequences obtained from Venezuelan (n = 3) and Colombian (n = 42) specimens were identified as *An*. *albitarsis* F by high homology (99.20–100%) with sequences deposited in the GenBank (S1 Table) and the BOLD Identification System. The newly generated sequences were submitted to the GenBank database under the accession numbers [MW136004- MW136048].

The overall GC content was 32.0%; 43 variable polymorphic sites, and 20 parsimony informative sites were recorded. High *MT-CO1* haplotype diversity (Hd = 0.990 ± 0.007 S.D.) and low nucleotide diversity (π = 0.012 ± 0.001 S.D.) were detected (Table 1). The overall mean genetic diversity (K2P) was 0.013 and the average genetic distance between populations from Colombia and Venezuela corresponded to 0.014. The highest genetic divergence (0.016) was found between localities from Venezuela (Guárico/Calabozo [GCA]; Bolívar/San Rafael/ [BSR]) and Colombia, Meta/Puerto Gaitán (MPG), while the lowest genetic divergence (0.012) occurred between the Colombian localities MPG and San José del Guaviare/Guaviare (SJG). From 37 haplotypes, closely related between Venezuelan localities and VPC-Colombia, 31 (83.78%) were singletons and 6 (16.22%) were shared, linked by single or few mutations (≤ 4) (S2 Table).

**Table 1:** Summary of *MT-CO1* sequences datasets statistics.

Summary statistics^a^	*Anopheles albitarsis* F barcode cluster	*Anopheles albitarsis* complex four species barcode clusters reported in the North of South America^b^	*Anopheles albitarsis* complex nine species barcode clusters reported in South America^c^
No. of sequences (N)	45	168	100
No. of polymorphic sites (S)	43	89	92
Total No. of mutations (Eta)	44	98	103
No. of haplotypes (H)	37	106	75
Haplotype diversity (Hd) ± S.D.	0.990 ± 0.007	0.985 ± 0.004	0.991 ± 0.004
Nucleotide diversity (*π*) ± S.D.	0.012 ± 0.001	0.020 ± 0.001	0.034 ± 0.001
Average nucleotide differences (K)	6.192	10.370	17.312

^a^ Total number of sites (excluding sites with gaps/missing data): 513

^b^ *An*. *marajoara*; *An*. *janconnae*; *An*. *albitarsis* F; *An*. *albitarsis* I.

^c^ *An*. *albitarsis* s.s.; *An*. *oryzalimnetes*; *An*. *marajoara*; *An*. *deaneorum*; *An*. *janconnae*; *An*. *albitarsis* F; *An*. *albitarsis* G; *An*. *albitarsis* H; *An*. *albitarsis* I.

### Phylogenetic analyses based on barcode *MT-CO1* partial sequences

**Fig 1:**
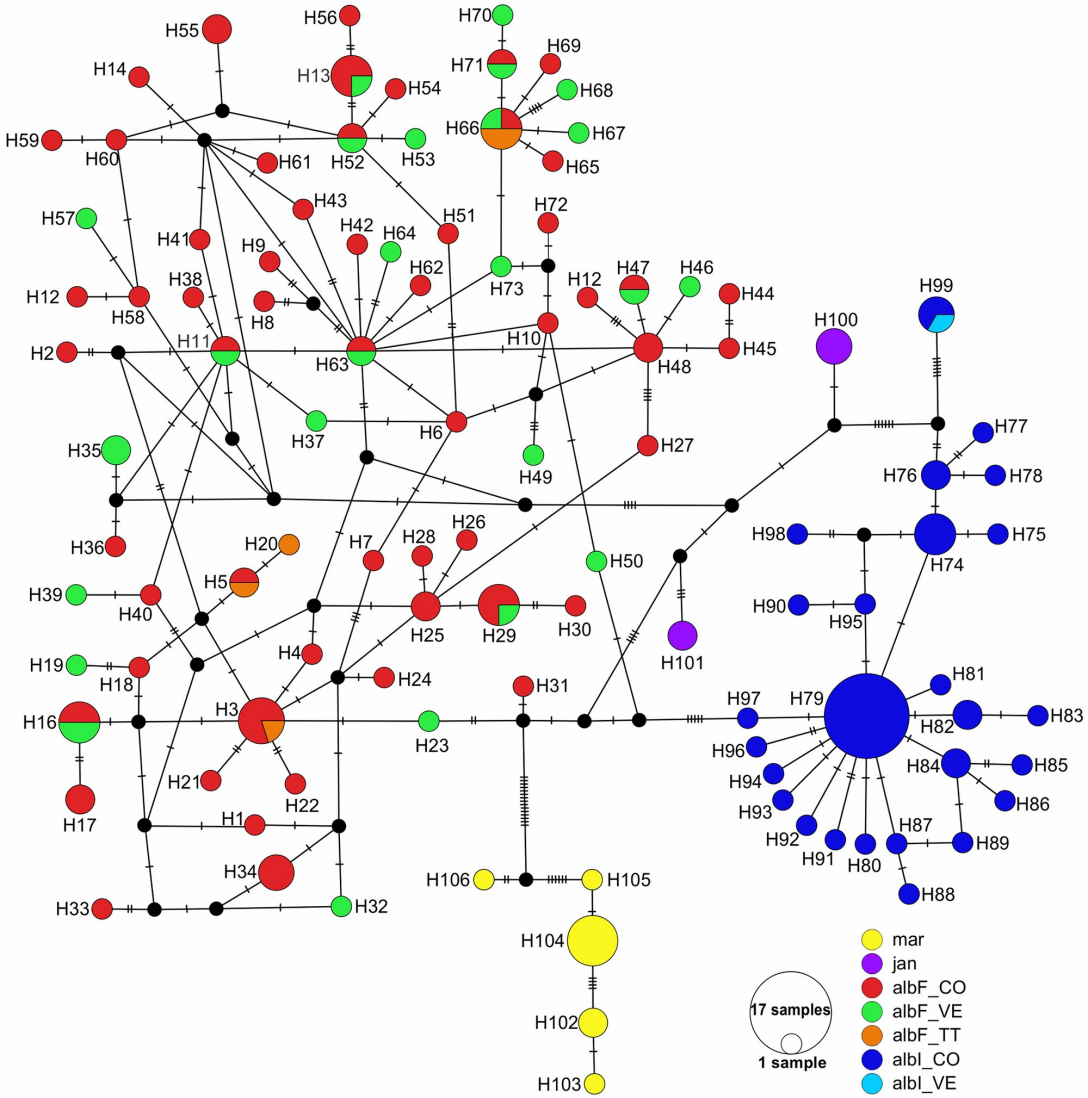
Genealogical analyses of *Anopheles albitarsis* complex species reported in the north of South America. Haplotype network of 106 *MT-CO1* haplotypes generated with TCS algorithm from 168 sequences dataset. Each taxa specimen and country are represented by a different color. The circle sizes indicate the frequency of individuals observed in each haplotype and, each notch on the links represents a mutated nucleotide position. The black solid circle indicates inferred missing intermediate steps between observed haplotypes (single nucleotide changes). **mar**: *An*. *marajoara*; **jan**: *An*. *janconnae*; **albF**: *An*. *albitarsis* F; **albI**: *An*. *albitarsis* I. **CO**: Colombia, **VE**: Venezuela, **TT**: Trinidad. *This analysis included all available sequences for *An*. *albitarsis* F and *An*. *albitarsis* I in the Genbank and the BOLD Systems database.

The dataset for the first analysis with *An*. *albitarsis* F, *An*. *albitarsis* I, *An*. *marajoara* and *An*. *janconnae* sequences from specimens collected in northern South America (N = 168) showed an overall GC of 32.2%, 89 variable polymorphic sites and 57 parsimony informative sites. The overall mean genetic distance (K2P) was 0.023, the average intra-specific genetic divergence was 0.009 (range: 0.006–0.013) and the average inter-specific divergence was 0.040 (range: 0.026–0.055). The most genetically divergent clusters were *An*. *marajoara* with *An*. *janconnae* (0.055), while lowest genetic divergence was found in *An*. *albitarsis* F with *An*. *albitarsis* I (0.026). For these taxa, a high *MT-CO1* haplotype diversity (Hd = 0.985 ± 0.004 S.D.) and low nucleotide diversity (π = 0.020 ± 0.001 S.D.) were also detected (Table 1). A total of 106 haplotypes were calculated: 79 (74.53%) were singleton and 27 (25.47%) shared haplotypes linked by a single or a few mutations (≤ 6) (Fig 1, S3 Table). The largest haplotype of *An*. *albitarsis* I (H79) was represented with 17 sequences from the Caribbean region of Colombia while the haplotypes H1 to H73 corresponded to *An*. *albitarsis* F where H66 was the most widely distributed with sequences from Vichada/Colombia, Bolívar (Jabillal)/Venezuela and St. George/Trinidad. Colombia shared more haplotypes (H11, H13, H16, H29, H47, H52, H63 and H71) with Venezuela than with Trinidad (H3, H5). The highest number of mutational steps was found between *An*. *albitarsis* F with *An*. *marajoara*. The Neighbor Joining (NJ-K2P) and Bayesian inference (BI) trees revealed *An*. *marajoara* as the basal clade and *An*. *albitarsis* F as the most recent clade. However, NJ-K2P grouped a clade for three sister taxa (BSV = 80%): *An*. *albitarsis* F (BSV = 64%) closer to *An*. *albitarsis* I (BSV = 78%) and *An*. *janconnae* (BSV = 99%); meanwhile the BI tree showed sister taxa *An*. *albitarsis* F closer to *An*. *albitarsis* I but poorly supported (BPP = 0.56).

**Fig 2:**
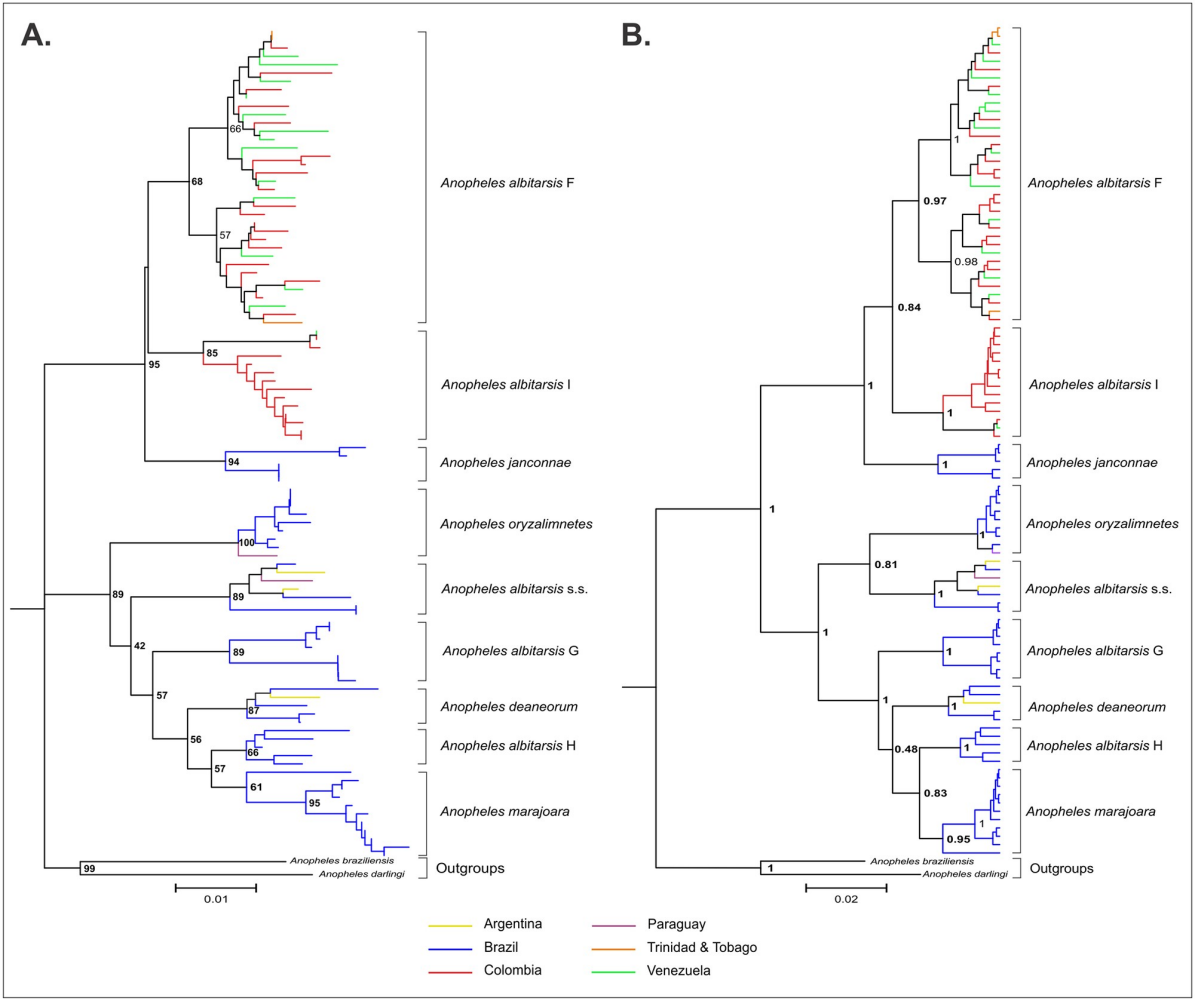
Phylogeny of *Anopheles albitarsis* complex in South America. **A**. Bootstrapped NJ tree based on 1000 replicates of K2P data matrices from 100 sequences dataset. **B**. Bayesian inference tree based on posterior probabilities. Outgroup taxa include *An*. *darlingi* (GenBank: DQ076236) and *An*. *braziliensis* (GenBank: DQ076238). Color-coding represents the country of origin of *MT-CO1* sequences taxa.

**Fig 3:**
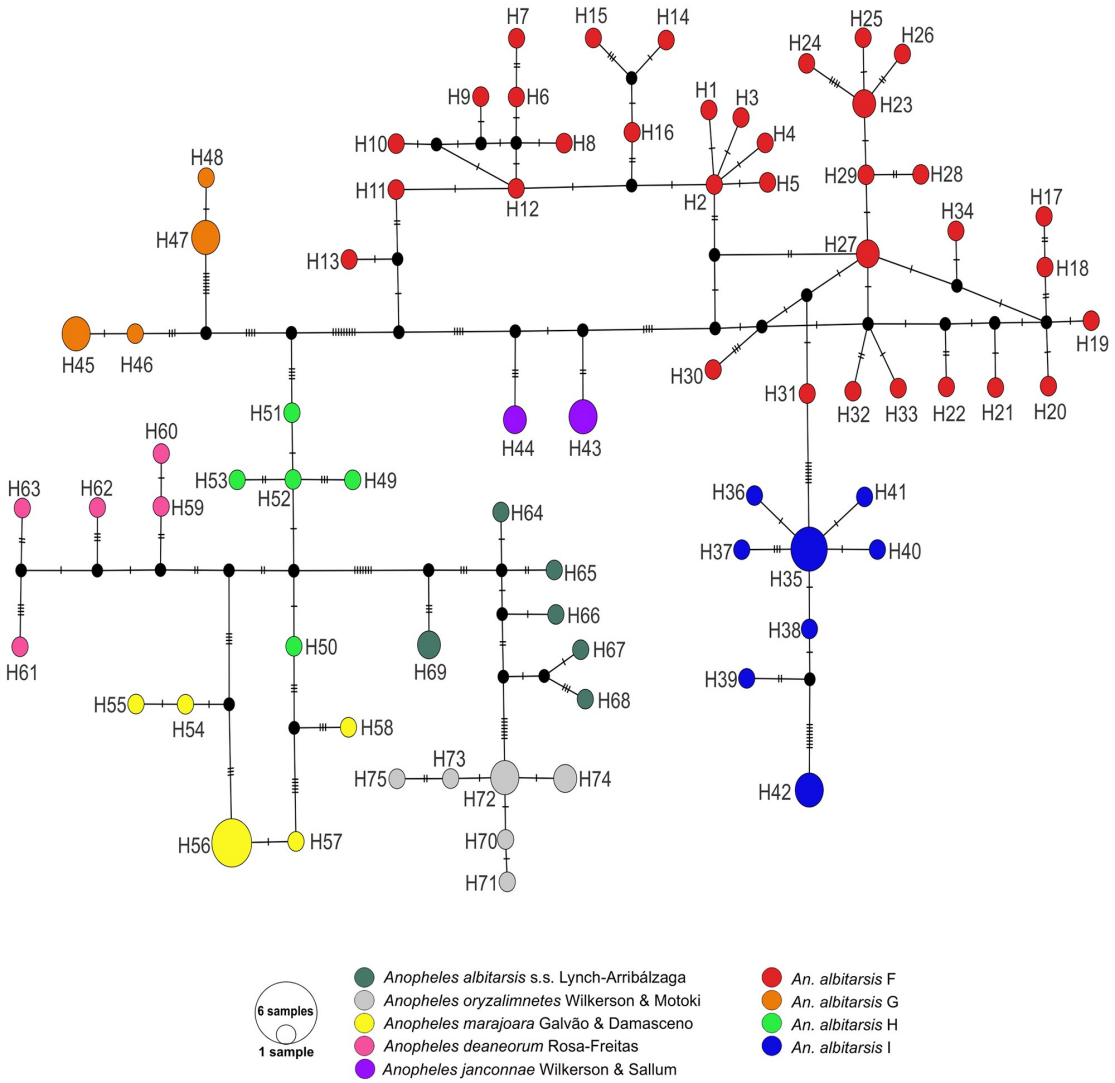
Genealogical analyses for nine species of the *Anopheles albitarsis* complex in South America. Haplotype network of 75 *MT-CO1* haplotype generated with TCS algorithm from 100 sequences dataset. The circles represent individual haplotypes with size proportional to frequency and, each notch on the links represents a mutated nucleotide position. The black solid circle indicates inferred missing intermediate steps between observed haplotypes (single nucleotide changes).

The second analysis with sequences (N = 100) representing all members of the Albitarsis Complex generated a fragment length of 658bp, with a large region of overlap located at the positions 1462 to 2120 of the *An*. *albitarsis* s.s. mitochondrion complete genome (GenBank: HQ335344) excluding the primer regions. The average nucleotide composition percentages for this dataset were A = 29.3%, C = 15.8%, G = 16.3% and T = 38.6%; GC content = 31.2% with 92 variable polymorphic sites and 67 parsimony informative sites. The overall mean genetic distance (K2P) was 0.036, the average intra-specific genetic divergence was 0.010 (0.004–0.014) and the average inter-specific divergence corresponded to 0.040 (0.021–0.055). The most genetically divergent clusters were *An*. *marajoara* with *An*. *janconnae* (0.055) followed by *An*. *marajoara* with *An*. *albitarsis* F and *An*. *albitarsis* G with *An*. *janconnae* (identical values respectively 0.052). Most similar clusters were *An*. *marajoara* with *An*. *albitarsis* H (0.021). The Neighbor Joining (NJ-K2P) and Bayesian inference (BI) trees were represented with nine distinct clusters (Fig 2) and similar topologies. The NJ-K2P tree showed highly supported clades for all taxa in the Albitarsis Complex (BSV: 85–100%) and the BI tree showed robust probabilities (BPP: 0.95–1) for all terminal taxa. Two main clades were clearly identified; *An*. *albitarsis* F was located at the clade 1 with *An*. *albitarsis* I and *An*. *janconnae*. The *MT-CO1* haplotype diversity (Hd = 0.991 ± 0.004 S.D.) and nucleotide diversity (*π* = 0.034 ± 0.001 S.D.) showed a similar pattern with the other analyses performed in this study (Table 1). The TCS network generated 75 haplotypes, 62 (82.67%) singletons and 13 (17.33%) were shared. Each of the clades identified in the phylogenetic trees was well-recognizable in the haplotypes network (Fig 3). *Anopheles albitarsis* F had the largest number of haplotypes (H1 –H34); *An*. *albitarsis* I (H35) and *An*. *marajoara* (H56) were the most shared haplotypes, including sequences from neighboring locations (S4 Table).

DNA barcodes showed not overlapping intra and interspecific divergences. This revealed a barcoding gap (threshold 0.014–0.021) between the largest intraspecific and smallest interspecific mean genetic distances (S1 Fig). The exhibiting interspecific divergence (≥2%) suggested distinct lineages within the Albitarsis Complex.

### Published records of geographic distribution and bionomics of *Anopheles albitarsis* s.l., north of the Amazon River

**Fig 4:**
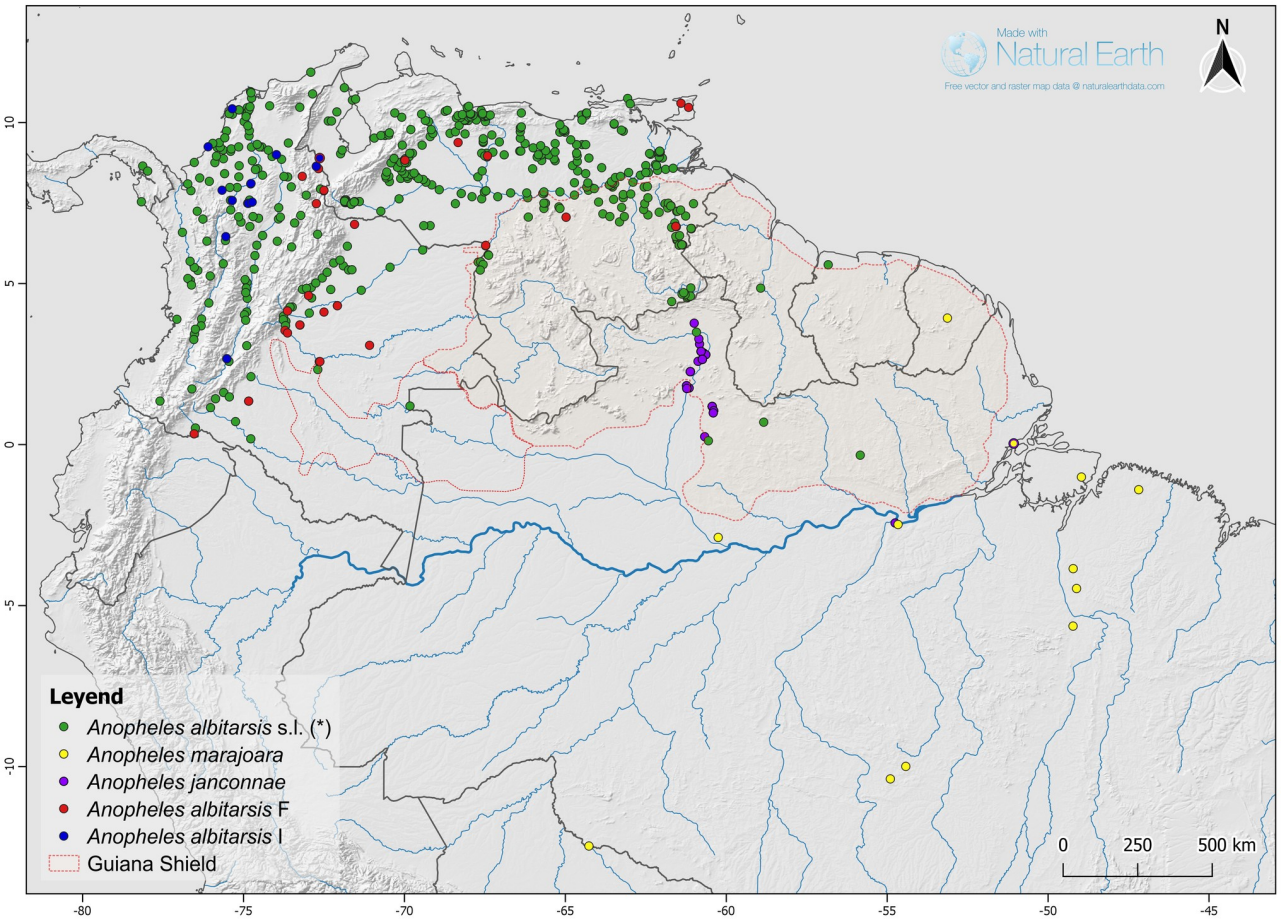
Geographic distribution of *Anopheles albitarsis* complex species in the north of South America. Note the distribution above the Amazon River of four species within the *Anopheles albitarsis* complex. (*) Species previously reported as *An*. *albitarsis*, *An*. *marajoara* or *An*. *allopha* were included as *An*. *albitarsis* s.l. The map was created in QGIS v 3.4 [59] using free vector and raster map data obtained from Natural Earth (public domain): naturalearthdata.com for illustrative purposes.

Formal records of *An*. *albitarsis* F based on DNA *MT-CO1* barcode, nDNA *white* gene, microsatellites and rDNA-ITS2 sequences showed a geographic distribution north of the Amazon river with reports from Colombia, the island of Trinidad, and Venezuela [9, 11, 13, 14, 16, 17, 21] (Fig 4).

In Colombia, *An*. *albitarsis* F (as *An*. *marajoara*, *An*. *albitarsis* s.l., *An*. *allopha*) corresponds to a gene pool associated with the Orinoquia Region (trans-Andean genetic pool) with reports from the Departments located east of the Andean Eastern Cordillera: Caquetá, Guaviare, Meta, Vichada [9, 15, 16, 21, 34], Putumayo [60], Casanare and Arauca [23] up to Norte de Santander near the border with Venezuela [16, 23]. Because local populations have been found naturally infected by *P*. *falciparum* [21, 22], and have been successfully infected by *P*. *vivax* in controlled conditions [61], this species has been considered a regional and opportunistic vector that maintains the transmission of malaria parasites throughout the year together with *An*. *darlingi* [15, 21]. Interestingly, Jimenez et al. [21] reported an Entomological Inoculation Rate (EIR) of 5.1 biting indoors and outdoors between 1800 and 1900 hrs in urban and peri urban neighborhoods of Puerto Carreño, Vichada. These authors also reported that this species is more active outdoors after sunset until 2100 hrs with a biting peak between 1800 and 1900 hrs, and a biting rate of 2.57 bites/person/hour [21]. The species has also been collected in urban areas of Villavicencio, Meta where it was more abundant outdoors with a biting rate of 11 bites/man/hour while indoors the biting rate was much lower (1.55); in rural areas the outdoor biting rate was 1.55 [62]. *Anopheles albitarsis* F (as *An*. *marajoara*) have been collected from a wide variety of larval habitats such as springs, streams, *morichales* (a flooded savannah area where the dominant tree is the palm *Mauritia flexuosa* or *moriche*) and lagoons; distinctly it has been also collected from man-made larval habitats exposed to sunlight such as fish ponds, wells and puddles [62].

*Anopheles albitarsis* I (as *An*. *albitarsis* s.l., *An*. *marajoara*) has been reported from the inter-Andean valleys of Colombia in the Departments of Antioquia and Huila [11, 15, 16, 23, 24, 63], and from the lowlands of the northern Departments of Bolívar, Córdoba and Magdalena [16, 23]; interestingly, in Norte de Santander Department, Colombia and Zulia State, Venezuela, this species occurs sympatrically with *An*. *albitarsis* F [11, 24]. This species has been collected at sunset in urban areas of El Banco, Magdalena and in Cáceres, Antioquia [15]. In general *An*. *albitarsis* I has been found in lowland dry and humid interior forests, strongly affected by human interventions as gold mining, cattle ranching, logging and small scale agriculture [16, 23, 64–69]. Larvae have been collected in sunlit flooded pasture fields, fish ponds and ponds [63, 66]; adult abundance was low (< 1%) [67, 68] except in some localities of the Caucasia Municipality, Antioquia, where up to 38.5% of mosquitoes collected on human landing catches were identified as *An*. *albitarsis* s.l. [69], biting mainly outdoors before midnight [67, 68]. So far *An*. *albitarsis* I has not been found positive for *Plasmodium* spp.; in these regions of Colombia the incriminated vectors are *An*. *darlingi*, *An*. *albimanus* Wiedemann and *An*. *nuneztovari* Gabaldon [64, 66–68].

In Venezuela, *An*. *albitarsis* F (as *An*. *albitarsis* s.l., *An*. *marajoara*) has a wide distribution; it has been reported from all the states at altitudes below 1000 m [17, 19, 25, 30, 31, 37, 70–81]. *Anopheles albitarsis* F immature stages have been found in rice fields, streams, swamps, *herbazales* (flooded savannahs), river margins, lagoons in forests and in savannahs, and pools in riverbeds during the dry season; forest lagoons are mainly the result of abandoned gold mines [37, 72, 74–76]. Larval habitats are usually located in sunlit open spaces, where the water color is amber and anopheline species are associated with grasses, aquatic vegetation such as green algae, *Utricularia* sp., *Ludwigia* sp., *Eleocharis* sp. and *Mayaca* sp. [74, 76]. This species was most abundant in lagoons and *herbazales* in August, three months after the peak of rains in gold mining areas of southern Venezuela [74]; adults showed no significant correlation with rainfall and were more abundant than *An*. *darlingi* all year round [73]. While in western Venezuela *An*. *albitarsis* F (as *An*. *albitarsis* s.l.) adults were significantly more abundant in August, one month after the peak of rains [79]. Longitudinal studies conducted in malaria endemic areas in western and southern Venezuela, as well as in the center of the country, the latter associated with rice fields, have shown a similar biting pattern along its range of distribution: it is active throughout the night indoors and outdoors and about 70% of bites occurred outdoors before midnight [37, 73, 79, 82–84] with preference of feeding on humans relative to bovines [85]. Along its range of distribution in Venezuela, as well as other species of the subgenus *Nyssorhynchus*, this species does not rest inside houses, being collected early morning resting on vegetation around houses [79, 83–85]. *Anopheles albitarsis* F (as *An*. *albitarsis* s.l.) has been found positive for malaria parasites in Venezuela in 1984 during an epidemic outbreak of *P*. *vivax* in Portuguesa State [37], where more recently Ruíz-López et al. [11] confirmed the presence of this lineage. This species is considered a secondary vector in western Venezuela where it was found positive for *P*. *vivax*-210 circumsporozoite protein [78], although during the dry season its vectorial capacity is similar to that of *An*. *nuneztovari* s.l., the principal malaria vector in the region [86]. In the malaria hotspot of southern Venezuela (Sifontes Municipality, Bolívar State), *An*. *albitarsis* F (as *An*. *marajoara*) has been found positive for circumsporozite protein of *P*. *falciparum*, *P*. *vivax*-247 and *P*. *vivax*-210 [30], with an overall sporozoite rate of 1.27 and entomological inoculation rate (EIR) of 1.25, while for *An*. *darlingi* the EIR was 2.21 [31]. Nevertheless, more recently [87] using nested PCR reported a marked increase of infection rates of anophelines in the hotspot of Sifontes Municipality where *An*. *albitarsis* F (as *An*. *albitarsis* s.l.) had higher infection rates than *An*. *darlingi*. Furthermore, the *An*. *albitarsis* F infection rate was 5.4%, followed by *An*. *darlingi* with 4.0 and *An*. *nuneztovari* s.l. with 0.5%. *Plasmodium vivax* accounted for 3.7% (61/1633) of infections in mosquitoes and 0.2% (4/1633) for *P*. *falciparum*. These authors concluded that the infection rate by *Plasmodium* spp. has increased more than three times in *An*. *albitarsis* F in ten years. Evaluations of susceptibility to insecticides conducted in 1994 and 2005 [88] have shown that populations from the Calabozo area, associated with rice crops, exhibit multiple resistance to the insecticides DDT, organophosphates and pyrethroids where the resistance mechanisms involved were increased level of nonspecific esterases, and evidence of insensitive acetylcholinesterase.

*Anopheles albitarsis* F (as *An*. *marajoara*) larvae have been collected in the island of Trinidad in rice fields, where they were more abundant during the rainy season [27]. Human landing catches between 1500 h and 2300 h showed a similar biting pattern indoors and outdoors with a peak of biting at 1900 hours [27, 89]. Exophilic resting behavior suggested that this species could play an important role in malaria transmission [89]. Nevertheless, the last outbreak of malaria in Trinidad and Tobago was reported in 1991 in villages located in the southwest tip of the island of Trinidad and the incriminated vector was *An*. (*Nys*.) *aquasalis* Curry [90]; until now *An*. *albitarsis* F has not been incriminated as a vector of malaria parasites in Trinidad and Tobago.

There are no recent published reports of *An*. *albitarsis* s.l. from Guyana. Giglioli [91] reported *An*. *albitarsis* from the savannah region of East Demerara and West Coast Berbice; Linthicum [36] examined specimens of *An*. *marajoara* collected by Aitken in 1962 in Georgetown, Berbice and Corentyne, and the most recent report is that of Rambajan [92] of *An*. *albitarsis* s.l. (as *An*. *allopha*) from coastal and interior parts of the country. The same seems to be the situation for Suriname; in fact, Hudson [93] reported *An*. *albitarsis* s.l. (as *An*. *allopha*) in New Nicherie associated with rice fields and Van der Kuyp [94] collected *An*. *albitarsis* in coastal areas. Apart from these reports *An*. *albitarsis* s.l. has not been collected in Suriname in over 30 years. However, in French Guiana, *An*. *marajoara* was confirmed by DNA *MT-CO1* barcoding associated with an outbreak of *P*. *vivax* malaria in gold mining camps in forested areas [95, 96].

Linthicum [36] and Sinka et al. [57] revisions included reports of *An*. *marajoara* from Costa Rica and Panamá. There are no recent records from Costa Rica. The reports date from specimens collected by Komp in 1936, 1941 and 1942 [36]. Reports from Panamá are mainly from the Canal Zone by Baxter & Zetek [97], Arnett [98] and Blanton & Payton [99]. Loaiza et al. [100] provided an update on the distribution of anophelines from Panamá based on data collected between 1970 and 2005 by the personnel of the Ministry of Health, reporting *An*. *albitarsis* s.l. from the western coast of the Darien malaria endemic area; no DNA sequences are available to confirm the species within the Albitarsis Complex in Panamá.

The Albitarsis Complex has a wide distribution in Brazil and extends south to Bolivia, Paraguay and Argentina [11]; so far it has not been reported from Ecuador or Perú despite there being a record of *An*. *albitarsis* F from Putumayo Department in Colombia [60] on the border with Ecuador. In the states of northern Brazil, parts of which lie on the Guayana Shield, four species have been reported: *An*. *marajoara*, *An*. *oryzalimnetes*, *An*. *janconnae* and *An*. *albitarsis* G [11, 13]. Presently, the only species of the Albitarsis Complex identified in the State of Roraima, which borders with Venezuela, has been *An*. *janconnae* [11, 13, 101]. Collections of immature stages and adults around Boa Vista, have yield significantly large numbers of *An*. *janconnae* (as *An*. *albitarsis* E) compared to *An*. *darlingi* and they are incriminated in malaria transmission with lower infection rates than *An*. *darlingi* [102, 103]. McKeon et al. [101] studied larval habitats in the savannah ecoregion of Roraima and characterized *An*. *janconnae* as a specialist species in terms of the types of larval habitats and its abiotic and biotic characteristics required; this species showed a positive correlation with water flow and a negative relationship to sun exposure. *Anopheles marajoara* and *An*. *oryzalimnetes* were found in lowland forests in Pará States, north of the Amazon River; *An*. *oryzalimnetes* was a specialist species preferring saline larval habitats exposed to sun light, while *An*. *marajoara* was found in diverse larval habitats and could not be classified as specialist [101, 103–105]. Although *An*. *albitarsis* G have been also collected in Pará State, south of the Amazon River [11], there is not available data on its bionomics and ecology. Also, in the State of Amapá, north of the Amazon River, *An*. *janconnae* and *An*. *marajoara* have been found sympatrically [11], but no data on their bionomics is available. Galardo et al. [106] reported that *An*. *marajoara* s.l. in Amapá was more abundant during the rainy season and positive for *P*. *vivax*; the species larval habitats were flooded forests and temporal or permanent open pastures. Nevertheless, it is not possible to determine to which species of the Complex this data is associated. So far, around Manaus, Amazonas State the only species confirmed is *An*. *albitarsis* G from forested areas [11, 107] but no information was provided in terms of abundance, biting rate, larval habitats and/or its medical importance.

## Discussion

In the present study, we considered that previous reports on *An*. *albitarsis* s.l., *An*. *allopha* and *An*. *marajoara* from Colombia, Venezuela, and the island of Trinidad referred to either *An*. *albitarsis* F or *An*. *albitarsis* I based on the previous studies [9, 11, 13, 15–17, 21, 23, 24]. Molecular analyses of DNA *MT-CO1* barcodes revealed the presence of *An*. *albitarsis* F in endemic malaria regions of Colombia (MPG, SJG and VPC) and Venezuela (GCA, BSR). The genetic distance between Venezuelan and Colombian populations was 1.2% with Meta population (MPG) as the most divergent. In contrast, the haplotype analysis showed Venezuelan localities more closely related to VPC, Colombia, located in the Orinoquia Region, bordering Venezuela (S2 Table). In fact, Puerto Carreño (VPC) is located at the confluence of the Meta and Orinoco rivers on the border with Venezuela which suggests that previous reports of *An*. *albitarsis* s.l. (as *An*. *marajoara*) from Apure [25] and Amazonas States [77], with similar altitude, vegetation and larval habitats, are actually *An*. *albitarsis* F. The present results support the broader distribution and public health importance of this taxon in northern South America, as indicated in earlier studies [9, 11, 15, 16]. Furthermore, the confirmation in this study of *An*. *albitarsis* F from the center of the country in Venezuela and in the gold mining area of Bolívar State, together with previous reports from other localities [11, 17] suggest that *An*. *albitarsis* F is present throughout the country where *An*. *albitarsis* s.l./*An*. *marajoara* has been reported from all the states and all ecoregions [19, 25, 70–88, 108]. Also, our findings and other reports [11, 15, 16, 21, 34, 60] suggest that *An*. *albitarsis* F is the only member of the Albitarsis Complex present in Colombia, east of the Eastern Andean Cordillera (trans-Andean genetic pool). *Anopheles albitarsis* F is the only species of the Albitarsis Complex present in the island of Trinidad [11] and it has not been reported from Ecuador, Perú and the island of Tobago; nevertheless, the confirmation of *An*. *albitarsis* F from Puerto Asís, Putumayo Department [60] on the Colombian border with Ecuador, suggests that this species might be also present in Ecuador since this is an active border with significant moving human population and similar ecology on both sides of the border. Also, this species shows rapid adaptation and expansion [14]. The absence of *An*. *albitarsis* F from the island of Tobago, located 35 km northeast of the island of Trinidad, might be due to its different geological origin, which resulted as in the rest of the Lesser Antilles, from the subduction of the South American Plate under the Caribbean Plate during the Mid-Eocene and Oligocene becoming a separate biogeographic region [109, 110]. As previously described in Results, this species is a generalist, exploiting different types of oviposition sites usually exposed to sunlight and associated with highly modify environments by human activities such as cattle ranching, agriculture and mining. It is important to point out that *An*. *albitarsis* F has not been collected in more pristine environments in Venezuela such as the Amerindian Yanomami territory of Amazonas State [111, 112] and the Amerindian Ye’kwana-Sanema territory along the Caura/Erebato rivers, Bolívar State [113], while it has been collected in Amazonas State around villages along the Orinoco river on the border with Colombia [77] and around *criollo* villages in the lower Caura river, Bolívar State [17, 76, 80, 82, 113]. Within its range of distribution, *An*. *albitarsis* F bites mainly outdoors before midnight [21, 62, 73, 79, 82, 83] and shows exophilic resting behavior [79, 85], characteristics that offer challenges for vector control since the traditional methods using long lasting treated nets (LLINS) and indoor residual spraying (IRS) are ineffective. *Anopheles albitarsis* F has been confirmed as a vector of malaria parasites in Colombia [21, 22] and Venezuela [30, 31, 78, 87]. Over the past 30 years the anarchic extensive deforestation and intensive human mobilization in Bolívar State, and specifically in the malaria hotspot of Sifontes municipality, southern Venezuela, has resulted in a change of species composition and abundance of anophelines. In fact, studies conducted in Sifontes between 1992–1997 showed that in general anopheline abundance was very low and the most frequent species collected was *An*. *darlingi* [114]; progressively it has been shown how species composition changed and abundance increased [72–75, 84]. Furthermore, a dramatic increase in infection rates of anophelines has been reported, where *An*. *albitarsis* F showed a higher infection than that of *An*. *darlingi*, until then the principal vector in the region. In fact, *An*. *albitarsis* F (as *An*. *albitarsis* s.l.) has more than tripled its human Plasmodia infection rates in ten years [31, 87]. This phenomenon supports the prediction models proposed for the year 2070 by Laporta et al. [115] where climatic and landscape effects might lead to shifts in the importance of roles and distribution of species of the Albitarsis Complex over *An*. *darlingi* for transmission caused by global warming. Similar observations were reported from northern Brazil for *An*. *janconnae* (as *An*. *albitarsis* s.l.). This species was significantly more abundant than *An*. *darlingi* in urban areas of Boa Vista, Roraima State, and was an important vector with higher EIR despite *An*. *darlingi* showed a higher infection rate [102, 103]. Meanwhile in Amapá State, Galardo et al. [106] reported higher sporozoite rates for *An*. *marajoara* s.l. in relation to *An*. *darlingi*; nevertheless, in this State *An*. *janconnae* and *An*. *marajoara* have been found sympatrically [11], and therefore it is not possible to determine to which species of the Complex these data are associated. *Anopheles marajoara* have been confirmed from some malaria foci in southern French Guiana [95] and incriminated as an important vector during an outbreak with a *P*. *vivax* infection rate of 6.4% while *An*. *darlingi* showed an infection rate of 1.1% for *P*. *falciparum* [96]. Intensive entomological surveys in Suriname have not reported the presence of *An*. *albitarsis* s.l. for over 30 years; a similar situation is found in Guyana, although entomological surveillance has not been as intense as in Suriname. Due to the shared border between Guyana (Essequibo) and Venezuela (Bolívar State), similar epidemiological and ecological conditions, associated with gold mining and dynamic movements of populations across the border, it is likely that *An*. *albitarsis* F is present and might be involved in the transmission of malaria parasites in Guyana.

In this study, phylogenetic relationship and genetic variation of *An*. *albitarsis* F, *An*. *albitarsis* I, *An*. *janconnae* and *An*. *marajoara* confirmed these taxa as separated lineages; based on interspecific divergences greater than 2% and distinct haplotype clusters for each species represented in a TCS network linked by multiple mutational steps (Fig 1). In terms of species differentiation, the haplotype network demonstrated that *An*. *albitarsis* F, *An*. *albitarsis* I and *An*. *janconnae* had relatively little divergence; *An*. *albitarsis* I corresponded to sequences from the Caribbean region of Colombia (GenBank: GQ153597—GQ153610), with a single record from Venezuela (Río Socuavo/Zulia, GenBank: JQ615189) that was in a shared haplotype with two records from Colombia (Tibú/Norte de Santander, GenBank: JQ615190; JQ615197). The haplotype (H79) was the largest cluster of sequences (N = 17) for the *An*. *albitarsis* complex phylogenetic analysis (Fig 1, S3 Table). A similar pattern occurred in both localities for the sister taxon *An*. *albitarsis* F that has been detected in sympatry and with low level of divergence [11, 13]. The high haplotype diversity and broad distribution encountered among haplotypes for *An*. *albitarsis* F in the present study and previously [11, 13] and the most widely distributed shared haplotype with sequences from Colombia, the island of Trinidad, and Venezuela could be explained by the correspondence of geographical proximity for the species and its sister clusters, either sympatric or overlapping with little ecological divergence [14]. However, further sampling is required to clarify these relationships.

*Anopheles albitarsis* I have been reported only from northwestern Venezuela and northeastern Colombia and extending south along the inter-Andean valleys between the Central and Eastern Andean Cordilleras (trans-Andean genetic pool) [11, 23]. This suggested that Colombian Andean Central and Western mountain ranges might limit the geographic distribution of *An*. *albitarsis* I to the west and south of the country. *Anopheles marajoara* (the basal species from which *An*. *albitarsis* F originated) and other members of the Albitarsis Complex have not been reported from Perú [11, 14]. It is proposed that *An*. *albitarsis* I is a lineage derived from *An*. *albitarsis* F. Apparently, *An*. *albitarsis* I is found in Venezuela only in sympatry with *An*. *albitarsis* F in Zulia State, north-eastern Venezuela near the border with Colombia. A similar situation was found in Norte de Santander Department [11]. Since *An*. *albitarsis* F and *An*. *albitarsis* I are in sympatry in the northern zone of the border between Colombia and Venezuela, this might be a zone of hybridization for the two lineages or a recent hotspot of speciation for them [15, 16]. There are no reports of *An*. *albitarsis* I from Trinidad and Tobago or elsewhere. Although *An*. *albitarsis* s.l. has been reported from Panamá [100], there are no sequences available; taking into account the current geographical distribution proposed for the clade of *An*. *albitarsis* F, *An*. *janconnae* and *An*. *albitarsis* I, it is suggested that *An*. *albitarsis* s.l. from Panamá could correspond to *An*. *albitarsis* I, a lineage more adapted to high temperatures, high relative humidity, and with more plasticity to occupy a wide range of larval habitats [16, 23, 64–66, 68, 69]. It is important to point out that so far, *An*. *albitarsis* I has not been found positive for *Plasmodium* spp. from either Colombia or Panamá, probably due to its low biting rate in relation to the incriminated vectors *An*. *albimanus*, *An*. *darlingi* and *An*. *nuneztovari* s.l. [64, 66].

Foley et al. [14] explored the possibility of finding a phylogenetic signal to explain ecological and environmental divergence for the proposed phylogenetic relationships of the Albitarsis Complex [11] which incorporated climatic and ecological data from the geographic locations of the sequencies included. Their results showed the Clade 1 comprised of *An*. *albitarsis* F, *An*. *albitarsis* I, and *An*. *janconnae*, as in the present study, and Clade 2 (all other species) were separated by the Amazon River. Events of dispersion and colonization of new habitats by these species might be determined by ecological requirements for each taxon, together with processes of landscape fragmentation, climatic changes and natural barriers that contributed to their recent speciation [14]. Furthermore, using *MT-CO1* sequences from the present study and previous records for all the members of the Albitarsis Complex, the monophyly of the Albitarsis Complex was strongly supported by Bayesian analysis (BPP: 0.95–1) and nine distinct clusters of the Albitarsis Complex were recovered as in previous studies [11, 13]. We have found a well-supported clade of sister taxa with *An*. *albitarsis* F, *An*. *janconnae*, and *An*. *albitarsis* I, all geographically located north of the Amazon River. These species correspond to separated lineages with interspecific divergences greater than 2% and distinct haplotypes clusters [11, 13]. As previously described, these species have distinctive geographic distribution, ecology, bionomy and vector characteristics [14]. In this sense, geological studies based on radar image interpretation, sedimentology and radio carbon dating indicated that the Amazon River originated during the Plio-Pleistocene with significant landscape changes that resulted in speciation [116]. The Amazon River determines the separation of Clade 1 and Clade 2 of the Albitarsis Complex [11, 14]; it has been proposed that it represents a major barrier for gene flow [117, 118]. However, Santorelli et al. [119] have rejected the river barrier hypothesis based on species distribution of different taxonomic groups around the Madeira River, Brazil, suggesting that the river had functioned as a vicariance barrier only for a low percentage of the total species identified (<1%). These contrasting hypotheses suggest that novel analyses are necessary to explain species distribution around major rivers. In addition, further studies are required to elucidate the evolutionary and taxonomic status to understand speciation events and phylogenetic relationships between closely related vector and non-vector species, which would allow the targeting of important disease vector species groups and the development of novel genetic-based vector and pathogen control methods.

It has been estimated that the genus *Anopheles* originated in Western Gondwana [120] in what is now South America during the early Cretaceous period around 145 to 100 million years ago [121, 122]. For the Albitarsis Complex, a monophyletic group, it has been estimated that it diverged approximately 39 MYA from its ancestor *An*. *darlingi* [123], following a latitudinal migration and subsequent diversification [121, 124] and within the group the time divergence was estimated at 0.58–2.25 MYA [13]. In accordance with the current natural geographical distribution registered for the Albitarsis Complex, we hypothesize that the origin of *An*. *albitarsis* s.l. was in the Precambrian Guayana Shield in southern Venezuela as the result of vertical migrations triggered by glacial/interglacial alternations followed by dispersal [125]. Although Conn & Mirabello [126] considered that the ancestry of *An*. *albitarsis* s.l. was in Venezuela, they proposed that it was due to the interaction of Pleistocene refugia and Miocene-Pliocene marine incursion that determined the distribution pattern of *An*. *albitarsis* s.l. At present, based on paleoecological, palynological and molecular phylogenetic studies, reported evidence suggests that the large biodiversity in the *Pantepui* region was not the result of Pleistocene refuge [127–131], but is the result of complex ecological and evolutionary trends initiated by Neogene tectonic events and paleogeographical reorganization, and maintained by the action of Pleistocene climatic changes [132]. The Guayana Shield, located between the Orinoco and Amazon basins, is a vast area which extends over half of Venezuela, Guyana, Suriname, French Guiana, north of Brazil and a small portion of south-east Colombia. This region, one of the riches in biodiversity and endemism, is characterized by the presence of steep table mountains or *tepuis* separated by large areas of savannahs and rain forests. Mayr and Phelps [133] used the term *Pantepui* to designate the region located between the summits of the *tepuis* of the Venezuelan Guayana Shield, including the “Gran Sabana” (Guayana Highlands) and adjacent regions of Brazil and Guyana. Steyermark [134] broadened this denomination to include the “Gran Sabana”, the savannahs and bornhardts of the Venezuelan Amazonas State and the northeast region of Bolívar State. This author defined all this region as the ‘Pantepui Refuge’. The ecological differences between the highlands and lowlands of the Guayana Shield were established and it was proposed that the term *Pantepui* should be used for ecosystems of middle and high altitudes (approximately above 1200 m) [135–137]. Navarro et al. [138], based on Parsimony Analysis of Endemicity between immature Culicidae and their aquatic habitats in plants, concluded that the Venezuelan Guayana Shield was an ancestral center of speciation. Similar observations were made for other taxa. Furthermore, within the *Pantepui* 60% of the vascular plant species and 87% of the frog species are endemic [139–141]. It seems that the *Pantepui* had emigrational pathways by physically connected valleys and slopes that contributed to radiation of species under the influence of cooling-warming oscillation patterns. Downward altitudinal migrations and dispersion of species followed by fragmentation and isolation of natural populations resulted in adaptive radiation and speciation [129].

The Orinoquia region in Colombia and the western savannahs of Venezuela have a unimodal rainfall pattern with a short dry period between December-April and wet season the rest of the year which produce flooding of lowlands providing available larval habitats for mosquitoes, particularly for species such as *An*. *albitarsis* F, that prefer oviposition sites totally exposed to sunlight. During the dry season, fragmentation of these habitats forces adults to find blood sources, natural refuges and to colonize new ecotopes far away from the initial oviposition sites. Since this condition is repeated every year, it can contribute to the dispersion of natural populations of mosquitoes. It is hypothesized that *An*. *albitarsis* F could be a lineage that separated from the of origin of basal members of the Albitarsis Complex such as *An*. *marajoara*, which could find a refuge in the *Pantepui* as samples have been collected from the Department of Caquetá in Colombia [15, 16] and in Jabillal, Bolívar State, Venezuela [17], both located on the borders of the Guayana Shield. Clear differences found in *An*. *albitarsis* F for the rDNA-ITS2, nDNA (partial loss of an intron in the *white* gene) [9] and microsatellite gDNA [16] and, mtDNA (*MT-CO1* barcode region) [11, 13] could be interpreted as divergences associated with a recent speciation process. The Venezuelan Andes could be a barrier for the expansion of *An*. *albitarsis* F towards north western Venezuela and north-eastern Colombia, although the Yaracuy depression between the North-Central Cordillera and the Venezuelan Andes could have been a corridor facilitating the expansion of *An*. *albitarsis* F towards the depression of the Maracaibo Lake and into Colombia. The island of Trinidad was part of north-eastern Venezuela until the early Holocene period (about 7000/6000 BCE) when sea level increased by about 60 m [142]; this event clearly explains the presence of *An*. *albitarsis* F on this island where it found appropriate ecological niches and at present is associated to irrigated rice fields [27]. Following a similar pathway, the expansions of populations of *An*. *albitarsis* s.l. from the *Pantepui* as an ancestral niche could derive in *An*. *janconnae*, currently located in the States of Roraima and Pará, Brazil, in the southern border of the Guayana Shield [11, 101] about 230 km from the Venezuelan border, where it has been described as a specialist species in relation to the selection of larval habitats in moving and sunlight exposed waters [101].

Despite the criticism regarding the use of unique markers such as a DNA barcode for species identification [143, 144]; barcode region analysis has proved to be a more sensitive marker for discriminating between incipient or very recently separated species/lineages compared to rDNA (ITS2) and *white* gene for the Albitarsis Complex [11, 13, 23, 145]. Likewise, the findings of this study revealed that this DNA fragment was robust for separating all the species of the Albitarsis Complex and suggested that species had reciprocal monophyly according to the current taxonomy [11]. However standard limits between intra and inter-species divergence cannot be generalized to many groups of organisms, the reported “barcoding gap” in our analyses corresponded to an interspecific variation threshold of at least 2% suggested for delimit separated species [43, 146–149]. Although sampling limitations in Venezuela (n = 3) could have led to bias in genetic differentiation among populations and species delimitation [150, 151], the present study generated 45 new sequences of *An*. *albitarsis* F, and the addition of publicly available *MT-CO1* sequences (n = 123) belonging to the Albitarsis Complex from north of the Amazon River, resulted in robust analyses. Nevertheless, further studies on population genetics that include more samples from each of the analyzed localities as well as localities from the proposed geographic expansion of this species are required to elucidate the morphological and genetic variation of *An*. *albitarsis* F and sister species. This would thus contribute to strengthening the phylogenetic relationships, hypotheses of their origin, expansion, and geographic dispersion presented in this study, and address questions about its taxonomic status, which has not yet been formally described.

## Conclusions

Phylogenetic analysis showed a well-supported clade with *An*. *albitarsis* F, *An*. *janconnae* and *An*. *albitarsis* I geographically restricted to northern South America. Biogeographic analysis based on the current geographical distribution of these sister taxa allowed the hypothesis that its ancestor came from *An*. *marajoara* and colonized the *Pantepui* region and then migrated to western Venezuela and Colombia suffering speciation events to generate *An*. *albitarsis* F and then *An*. *albitarsis* I. Migration south of Venezuela produced *An*. *janconnae*. *Anopheles albitarsis* F is recorded in Colombia, Venezuela and the island of Trinidad while *An*. *albitarsis* I is recorded in Venezuela and Colombia showing a hybridization zone in the border of both countries in Norte de Santander Department and Zulia State. Natural infections by *P*. *vivax* and *P*. *falciparum* in Colombia and Venezuela and epidemiological incrimination constitute strong evidence of the important role of *An*. *albitarsis* F in the transmission of malaria parasites. Entomological surveillance for these species in Colombia, Venezuela, Trinidad and Tobago, and also in Guyana, Ecuador, Perú, and Panamá, are mandatory to update the geographic distribution, ecological and biological aspects and its possible role as a regional vector of malaria parasites.

## Supporting information

S1 Table Comparison between DNA *mitochondrially encoded cytochrome c oxidase I* (*MT-CO1*) gene sequences obtained in this study and those available in the GenBank database. **N°**: Numerical order of the sequences; **Code**: abbreviation of the sequences obtained; **Percentage of Identify**: Similarity of the sequences obtained in this study with the sequences available in the GenBank; **Comparison in BLASTn, Author**: References of the sequences compared; **Species**: Species identified; **Accession N°**: Accession number of the compared sequences.
(DOCX)

S2 Table Information of the 37 haplotypes generated with DNA *mitochondrially encoded cytochrome c oxidase I* (*MT-CO1*) gene sequences database from Colombia (n = 42) and Venezuela (n = 3). **H**: Haplotypes; **N°**: Absolute frequency of individuals observed in each haplotype. Within parentheses are the numbers of individuals observed for each haplotype in each locality. **CO**: Colombia, **VE**: Venezuela.
(DOCX)

S3 Table Information of 106 haplotypes generated with the 168 DNA *mitochondrially encoded cytochrome c oxidase I* (*MT-CO1*) gene sequences database. **H**: Haplotypes; **N°**: Absolute frequency of individuals observed in each haplotype. Within parentheses are the numbers of individuals observed for each haplotype in each locality. **BR**: Brazil, **CO**: Colombia, **TT**: Trinidad, **VE**: Venezuela.
(DOCX)

S4 Table Information of 75 haplotypes generated with the 100 DNA *mitochondrially encoded cytochrome c oxidase I* (*MT-CO1*) gene sequences database. **H**: Haplotypes; **N°**: Absolute frequency of individuals observed in each haplotype. Within parentheses are the numbers of individuals observed for each haplotype in each locality. **BR**: Brazil, **CO**: Colombia, **TT**: Trinidad, **VE**: Venezuela.
(DOCX)

S1 Fig Barcode analysis plots of *Anopheles albitarsis* complex species reported in South America. Barcode gap analysis of all species within the *Anopheles albitarsis* complex, plots are based in distance matrices of the clusters determined using NJ-K2P distances. Y-axis: genetic divergence and X-axis clusters. **alb**: *An*. *albitarsis* s.s.; **ory**: *An*. *oryzalimnetes*; **mar**: *An*. *marajoara*; **dea**: *An*. *deaneorum*; **jan**: *An*. *janconnae*; **albF**: *An*. *albitarsis* F; **albG**: *An*. *albitarsis* G; **albH**: *An*. *albitarsis* H; **albI**: *An*. *albitarsis* I.
(TIF)
