# Supplementary material for: Updating the bionomy and geographical distribution of Anopheles (Nyssorhynchus) albitarsis F: A vector of malaria parasites in northern South America
Source: PLoS One. 2021 Jun 17;16(6):e0253230. doi: 10.1371/journal.pone.0253230 (PMC8211218; doi:10.1371/journal.pone.0253230)
Supplement: S1 Table — N°: Numerical order of the sequences; Code: abbreviation of the sequences obtained; Percentage of Identify: Similarity of the sequences obtained in this study with the sequences available in the GenBank; Comparison in BLASTn, Author: References of the sequences compared; Species: Species identified; Accession N°: Accession number of the compared sequences. (DOCX) [file pone.0253230.s001.docx]

**S1 Table.** Comparison between DNA *mitochondrially encoded cytochrome c oxidase I* (*MT-CO1*) gene sequences obtained in this study and those available in the GenBank database.

Continue

| **Collected Samples** | | |  | **Percentage of identify** | **Comparison BLASTn** | | |
| --- | --- | --- | --- | --- | --- | --- | --- |
| **Nº** | **Code** | **Locality/Department or State** | **Accession Nº** |  | **Author** | **Species** | **Accession Nº** |
| 1 | MPGS031-2 | Puerto Gaitán/Meta | MW136004 | 99.52 | Rubio-Palis et al. (2013) | *An. albitarsis* F | KC555064 |
| 2 | MPGS090-27 | Puerto Gaitán/Meta | MW136005 | 100 | Ruiz-Lopez et al. (2012) | *An. albitarsis* F | JQ615020 |
| 3 | MPGS34-14 | Puerto Gaitán/Meta | MW136006 | 99.84 | Ruiz-Lopez et al. (2012) | *An. albitarsis* F | JQ615020 |
| 4 | MPGS44-2 | Puerto Gaitán/Meta | MW136007 | 99.68 | Ruiz-Lopez et al. (2012) | *An. albitarsis* F | JQ615026 |
| 5 | MPGS50-3 | Puerto Gaitán/Meta | MW136008 | 99.84 | Ruiz-Lopez et al. (2012) | *An. albitarsis* F | JQ615033 |
| 6 | SJG0014-1 | San José del Guaviare/Guaviare | MW136009 | 99.68 | Ruiz-Lopez et al. (2012) | *An. albitarsis* F | JQ615035 |
| 7 | SJG0025-5 | San José del Guaviare/Guaviare | MW136010 | 99.84 | Rubio-Palis et al. (2013) | *An. albitarsis* F | KC555068 |
| 8 | SJG0025-10 | San José del Guaviare/Guaviare | MW136011 | 99.68 | Rubio-Palis et al. (2013) | *An. albitarsis* F | KC555070 |
| 9 | SJG038A-1 | San José del Guaviare/Guaviare | MW136012 | 99.52 | Ruiz-Lopez et al. (2012) | *An. albitarsis* F | JQ615021 |
| 10 | SJG0064A-10 | San José del Guaviare/Guaviare | MW136013 | 99.84 | Ruiz-Lopez et al. (2012) | *An. albitarsis* F | JQ615035 |
| 11 | SJG0083A-2 | San José del Guaviare/Guaviare | MW136014 | 99.52 | Ruiz-Lopez et al. (2012) | *An. albitarsis* F | JQ615035 |
| 12 | SJG0115-4 | San José del Guaviare/Guaviare | MW136015 | 99.68 | Ruiz-Lopez et al. (2012) | *An. albitarsis* F | JQ615026 |
| 13 | SJG0116A-5 | San José del Guaviare/Guaviare | MW136016 | 99.84 | Ruiz-Lopez et al. (2012) | *An. albitarsis* F | JQ615033 |
| 14 | SJG1-10 | San José del Guaviare/Guaviare | MW136017 | 99.52 | Ruiz-Lopez et al. (2012) | *An. albitarsis* F | JQ615039 |
| 15 | SJG4-4 | San José del Guaviare/Guaviare | MW136018 | 99.68 | Ruiz-Lopez et al. (2012) | *An. albitarsis* F | JQ614999 |
| 16 | SJG5-4 | San José del Guaviare/Guaviare | MW136019 | 99.84 | Rubio-Palis et al. (2013) | *An. albitarsis* F | KC555068 |
| 17 | SJG6-7 | San José del Guaviare/Guaviare | MW136020 | 99.84 | Ruiz-Lopez et al. (2012) | *An. albitarsis* F | JQ615026 |
| 18 | SJG7-23 | San José del Guaviare/Guaviare | MW136021 | 99.68 | Ruiz-Lopez et al. (2012) | *An. albitarsis* F | JQ615011 |
| 19 | SJG19-6 | San José del Guaviare/Guaviare | MW136022 | 99.84 | Ruiz-Lopez et al. (2012) | *An. albitarsis* F | JQ615033 |
| 20 | SJG19-35A | San José del Guaviare/Guaviare | MW136023 | 99.68 | Rubio-Palis et al. (2013) | *An. albitarsis* F | KC555063 |
| 21 | SJG28-3 | San José del Guaviare/Guaviare | MW136024 | 99.68 | Ruiz-Lopez et al. (2012) | *An. albitarsis* F | JQ615026 |
| 22 | SJG30-2 | San José del Guaviare/Guaviare | MW136025 | 99.19 | Rubio-Palis et al. (2013) | *An. albitarsis* F | KC555068 |
| 23 | VPC0001-4 | Puerto Carreño/Vichada | MW136026 | 99.68 | Rubio-Palis et al. (2013) | *An. albitarsis* F | KC555070 |
| 24 | VPC0071-5 | Puerto Carreño/Vichada | MW136027 | 99.52 | Ruiz-Lopez et al. (2012) | *An. albitarsis* F | JQ615020 |
| 25 | VPC0096-4 | Puerto Carreño/Vichada | MW136028 | 99.85 | Ruiz-Lopez et al. (2012) | *An. albitarsis* F | JQ615002 |
| 26 | VPC0104-4 | Puerto Carreño/Vichada | MW136029 | 99.84 | Ruiz-Lopez et al. (2012) | *An. albitarsis* F | JQ615013 |
| 27 | VPC0104-5 | Puerto Carreño/Vichada | MW136030 | 99.68 | Rubio-Palis et al. (2013) | *An. albitarsis* F | KC555070 |
| 28 | VPC0121-58 | Puerto Carreño/Vichada | MW136031 | 99.68 | Ruiz-Lopez et al. (2012) | *An. albitarsis* F | JQ615035 |
| 29 | VPC0129-9 | Puerto Carreño/Vichada | MW136032 | 99.68 | Ruiz-Lopez et al. (2012) | *An. albitarsis* F | JQ615020 |
| 30 | VPC0130-12 | Puerto Carreño/Vichada | MW136033 | 99.36 | Rubio-Palis et al. (2013) | *An. albitarsis* F | KC555068 |
| 31 | VPC0133-10 | Puerto Carreño/Vichada | MW136034 | 99.52 | Ruiz-Lopez et al. (2012) | *An. albitarsis* F | JQ615002 |
| 32 | VPC0134-8 | Puerto Carreño/Vichada | MW136035 | 99.70 | Ruiz-Lopez et al. (2012) | *An. albitarsis* F | JQ615035 |
| 33 | VPC0135-3 | Puerto Carreño/Vichada | MW136036 | 99.84 | Ruiz-Lopez et al. (2012) | *An. albitarsis* F | JQ615011 |
| 34  Continue | VPC0141-15 | Puerto Carreño/Vichada | MW136037 | 100 | Rubio-Palis et al. (2013) | *An. albitarsis* F | KC555068 |
| 35 | VPC0145-1 | Puerto Carreño/Vichada | MW136038 | 99.52 | Ruiz-Lopez et al. (2012) | *An. albitarsis* F | JQ615002 |
| 36 | VPC0147-6 | Puerto Carreño/Vichada | MW136039 | 99.52 | Ruiz-Lopez et al. (2012) | *An. albitarsis* F | JQ615020 |
| 37 | VPC0165-13 | Puerto Carreño/Vichada | MW136040 | 100 | Ruiz-Lopez et al. (2012) | *An. albitarsis* F | JQ614999 |
| 38 | VPC0177-7 | Puerto Carreño/Vichada | MW136041 | 99.52 | Ruiz-Lopez et al. (2012) | *An. albitarsis* F | JQ615020 |
| 39 | VPC0198-11 | Puerto Carreño/Vichada | MW136042 | 99.84 | Rubio-Palis et al. (2013) | *An. albitarsis* F | KC555070 |
| 40 | VPC9-2 | Puerto Carreño/Vichada | MW136043 | 99.52 | Ruiz-Lopez et al. (2012) | *An. albitarsis* F | JQ615033 |
| 41 | VPC11-5 | Puerto Carreño/Vichada | MW136044 | 99.52 | Ruiz-Lopez et al. (2012) | *An. albitarsis* F | JQ615002 |
| 42 | VPC17-6 | Puerto Carreño/Vichada | MW136045 | 100 | Ruiz-Lopez et al. (2012) | *An. albitarsis* F | JQ615012 |
| 43 | VE-GUA-CAL-00 | Calabozo/Guárico | MW136046 | 99.81 | Ruiz-Lopez et al. (2012) | *An. albitarsis* F | JQ615035 |
| 44 | VE-BOL-LC-1B | San Rafael/Bolívar | MW136047 | 99.26 | Ruiz-Lopez et al. (2012) | *An. albitarsis* F | JQ614999 |
| 45 | VE-BOL-LC-11B | San Rafael/Bolívar | MW136048 | 99.81 | Ruiz-Lopez et al. (2012) | *An. albitarsis* F | JQ615012 |

**Nº:** Numerical order of the sequences; **Code:** abbreviation of the sequences obtained; **Percentage of Identify:** Similarity of the sequences obtained in this study with the sequences available in the GenBank; **Comparison in BLASTn, Author:** References of the sequences compared; **Species:** Species identified.
