# Supplementary material for: Updating the bionomy and geographical distribution of Anopheles (Nyssorhynchus) albitarsis F: A vector of malaria parasites in northern South America
Source: PLoS One. 2021 Jun 17;16(6):e0253230. doi: 10.1371/journal.pone.0253230 (PMC8211218; doi:10.1371/journal.pone.0253230)
Supplement: S2 Table — H: Haplotypes; N°: Absolute frequency of individuals observed in each haplotype. Within parentheses are the numbers of individuals observed for each haplotype in each locality. CO: Colombia, VE: Venezuela. (DOCX) [file pone.0253230.s002.docx]

**S2 Table.** Information of the 37 haplotypes generated with DNA *mitochondrially encoded cytochrome c oxidase I* (*MT-CO1*) gene sequences database from Colombia (n=42) and Venezuela (n=3).

| **H** | **Nº** | **Species** | **Locality/** **Department or State** | **Country** |
| --- | --- | --- | --- | --- |
| **H1** | 1 | *An. albitarsis* F | San Rafael/Bolívar | VE |
| **H2** | 1 | *An. albitarsis* F | Puerto Carreño/Vichada | CO |
| **H3** | 1 | *An. albitarsis* F | San José del Guaviare/Guaviare | CO |
| **H4** | 1 | *An. albitarsis* F | San Rafael/Bolívar | VE |
| **H5** | 1 | *An. albitarsis* F | Puerto Carreño/Vichada | CO |
| **H6** | 1 | *An. albitarsis* F | San José del Guaviare/Guaviare | CO |
| **H7** | 1 | *An. albitarsis* F | Puerto Carreño/Vichada | CO |
| **H8** | 1 | *An. albitarsis* F | San José del Guaviare/Guaviare | CO |
| **H9** | 1 | *An. albitarsis* F | San José del Guaviare/Guaviare | CO |
| **H10** | 1 | *An. albitarsis* F | Puerto Carreño/Vichada | CO |
| **H11** | 2 | *An. albitarsis* F | Puerto Carreño/Vichada (2) | CO |
| **H12** | 1 | *An. albitarsis* F | Puerto Carreño/Vichada | CO |
| **H13** | 1 | *An. albitarsis* F | San José del Guaviare/Guaviare | CO |
| **H14** | 1 | *An. albitarsis* F | San José del Guaviare/Guaviare | CO |
| **H15** | 2 | *An. albitarsis* F | San José del Guaviare/Guaviare (2) | CO |
| **H16** | 1 | *An. albitarsis* F | Puerto Carreño/Vichada | CO |
| **H17** | 1 | *An. albitarsis* F | San José del Guaviare/Guaviare | CO |
| **H18** | 1 | *An. albitarsis* F | Puerto Carreño/Vichada | CO |
| **H19** | 1 | *An. albitarsis* F | Puerto Carreño/Vichada | CO |
| **H20** | 1 | *An. albitarsis* F | Puerto Carreño/Vichada | CO |
| **H21** | 1 | *An. albitarsis* F | San José del Guaviare/Guaviare | CO |
| **H22** | 1 | *An. albitarsis* F | San José del Guaviare/Guaviare | CO |
| **H23** | 1 | *An. albitarsis* F | Calabozo/Guárico | VE |
| **H24** | 2 | *An. albitarsis* F | Puerto Carreño/Vichada (2) | CO |
| **H25** | 1 | *An. albitarsis* F | Puerto Carreño/Vichada | CO |
| **H26** | 1 | *An. albitarsis* F | Puerto Carreño/Vichada | CO |
| **H27** | 1 | *An. albitarsis* F | Puerto Gaitán/Meta | CO |
| **H28** | 2 | *An. albitarsis* F | Puerto Gaitán/Meta (2) | CO |
| **H29** | 1 | *An. albitarsis* F | San José del Guaviare/Guaviare | CO |
| **H30** | 1 | *An. albitarsis* F | San José del Guaviare/Guaviare | CO |
| **H31** | 1 | *An. albitarsis* F | Puerto Carreño/Vichada | CO |
| **H32** | 3 | *An. albitarsis* F | Puerto Carreño/Vichada (3) | CO |
| **H33** | 1 | *An. albitarsis* F | Puerto Gaitán/Meta | CO |
| **H34** | 1 | *An. albitarsis* F | San José del Guaviare/Guaviare | CO |
| **H35** | 1 | *An. albitarsis* F | San José del Guaviare/Guaviare | CO |
| **H36** | 3 | *An. albitarsis* F | Puerto Gaitán/Meta (1); San José del Guaviare/Guaviare (2) | CO |
| **H37** | 1 | *An. albitarsis* F | Puerto Carreño/Vichada | CO |

**H:** Haplotypes; **N°:** Absolute frequency of individuals observed in each haplotype. Within parentheses are the numbers of individuals observed for each haplotype in each locality. **CO:** Colombia, **VE:** Venezuela.
