# Supplementary material for: Updating the bionomy and geographical distribution of Anopheles (Nyssorhynchus) albitarsis F: A vector of malaria parasites in northern South America
Source: PLoS One. 2021 Jun 17;16(6):e0253230. doi: 10.1371/journal.pone.0253230 (PMC8211218; doi:10.1371/journal.pone.0253230)
Supplement: S3 Table — H: Haplotypes; N°: Absolute frequency of individuals observed in each haplotype. Within parentheses are the numbers of individuals observed for each haplotype in each locality. BR: Brazil, CO: Colombia, TT: Trinidad, VE: Venezuela. (DOCX) [file pone.0253230.s003.docx]

**S3 Table.** Information of 106 haplotypes generated with 168 DNA *mitochondrially encoded cytochrome c oxidase I* (*MT-CO1*) gene sequences database.

Continue

| **H** | **Nº** | **Species** | **Locality/Department or State** | **Country** |
| --- | --- | --- | --- | --- |
| **H1** | 1 | *An. albitarsis* F | Arauquita/Arauca | CO |
| **H2** | 1 | *An. albitarsis* F | Arauquita/Arauca | CO |
| **H3** | 5 | *An. albitarsis* F | Arauquita/Arauca (1), Frederick Settlement/St. George (1), Puerto Asís/Putumayo (1), Puerto Gaitán/Meta (2) | CO/TT |
| **H4** | 1 | *An. albitarsis* F | Arauquita/Arauca | CO |
| **H5** | 2 | *An. albitarsis* F | Arauquita/Arauca (1), St. David/ St. Andrew (1) | CO/TT |
| **H6** | 1 | *An. albitarsis* F | Arauquita/Arauca | CO |
| **H7** | 1 | *An. albitarsis* F | Tibú/Norte de Santander | CO |
| **H8** | 1 | *An. albitarsis* F | Arauquita/Arauca | CO |
| **H9** | 1 | *An. albitarsis* F | Arauquita/Arauca | CO |
| **H10** | 1 | *An. albitarsis* F | Villanueva/Casanare | CO |
| **H11** | 2 | *An. albitarsis* F | Arauquita/Arauca (1), Rosa Blanca/Cojedes (1) | CO/VE |
| **H12** | 1 | *An. albitarsis* F | Villanueva/Casanare | CO |
| **H13** | 4 | *An. albitarsis* F | Tibú/Norte de Santander (3), Río Socuavo/Zulia (1) | CO/VE |
| **H14** | 1 | *An. albitarsis* F | Arauquita/Arauca | CO |
| **H15** | 1 | *An. albitarsis* F | Arauquita/Arauca | CO |
| **H16** | 4 | *An. albitarsis* F | Río Socuavo/Zulia (2), Puerto Carreño/Vichada (2) | VE/CO |
| **H17** | 2 | *An. albitarsis* F | Puerto Carreño/Vichada (2) | CO |
| **H18** | 1 | *An. albitarsis* F | Puerto Gaitán/Meta | CO |
| **H19** | 1 | *An. albitarsis* F | Jabillal/Bolívar | VE |
| **H20** | 1 | *An. albitarsis* F | St. David/St. Andrew | TT |
| **H21** | 1 | *An. albitarsis* F | Puerto Carreño/Vichada | CO |
| **H22** | 1 | *An. albitarsis* F | San José del Guaviare/Guaviare | CO |
| **H23** | 1 | *An. albitarsis* F | Jabillal/Bolívar | VE |
| **H24** | 1 | *An. albitarsis* F | San José del Guaviare/Guaviare | CO |
| **H25** | 2 | *An. albitarsis* F | San José del Guaviare/Guaviare (1), Puerto Carreño/Vichada (1) | CO |
| **H26** | 1 | *An. albitarsis* F | San José del Guaviare/Guaviare | CO |
| **H27** | 1 | *An. albitarsis* F | Puerto Carreño/Vichada | CO |
| **H28** | 1 | *An. albitarsis* F | Puerto Gaitán/Meta | CO |
| **H29** | 4 | *An. albitarsis* F | Puerto Gaitán/Meta (1), San José del Guaviare/Guaviare (2),  Rosa Blanca/Cojedes (1) | CO/VE |
| **H30** | 1 | *An. albitarsis* F | Puerto Carreño/Vichada | CO |
| **H31** | 1 | *An. albitarsis* F | Tibú/Norte de Santander | CO |
| **H32** | 1 | *An. albitarsis* F | Río Socuavo/Zulia | VE |
| **H33** | 1 | *An. albitarsis* F | Puerto Asís/Putumayo | CO |
| **H34** | 3 | *An. albitarsis* F | Puerto Carreño/Vichada (3) | CO |
| **H35** | 2 | *An. albitarsis* F | Río Socuavo/Zulia (2) | VE |
| **H36** | 1 | *An. albitarsis* F | Tibú/Norte de Santander | CO |
| **H37** | 1 | *An. albitarsis* F | Jabillal/Bolívar | VE |
| **H38** | 1 | *An. albitarsis* F | San José del Guaviare/Guaviare | CO |
| **H39** | 1 | *An. albitarsis* F | Río Socuavo/Zulia | VE |
| **H40** | 1 | *An. albitarsis* F | Puerto Carreño/Vichada | CO |
| **H41** | 1 | *An. albitarsis* F | Puerto Carreño/Vichada | CO |
| **H42** | 1 | *An. albitarsis* F | San José del Guaviare/Guaviare | CO |
| **H43** | 1 | *An. albitarsis* F | Cruce Granada/Meta | CO |
| **H** | **Nº** | **Species** | **Locality/Department or State** | **Country** |
| **H44** | 1 | *An. albitarsis* F | San José del Guaviare/Guaviare | CO |
| **H45** | 1 | *An. albitarsis* F | Puerto Carreño/Vichada | CO |
| **H46** | 1 | *An. albitarsis* F | Rosa Blanca/Cojedes | VE |
| **H47** | 2 | *An. albitarsis* F | Puerto Carreño/Vichada (1), Jabillal/Bolívar (1) | CO/VE |
| **H48** | 2 | *An. albitarsis* F | San José del Guaviare/Guaviare | CO |
| **H49** | 1 | *An. albitarsis* F | Río Socuavo/Zulia | VE |
| **H50** | 1 | *An. albitarsis* F | Jabillal/Bolívar | VE |
| **H51** | 1 | *An. albitarsis* F | San José del Guaviare/Guaviare | CO |
| **H52** | 2 | *An. albitarsis* F | Jabillal/Bolívar (1), Puerto Carreño/Vichada (1) | VE/CO |
| **H53** | 1 | *An. albitarsis* F | Río Boconó/Portuguesa | VE |
| **H54** | 1 | *An. albitarsis* F | Tibú/Norte de Santander | CO |
| **H55** | 2 | *An. albitarsis* F | Puerto Carreño/Vichada (2) | CO |
| **H56** | 1 | *An. albitarsis* F | Tibú/Norte de Santander | CO |
| **H57** | 1 | *An. albitarsis* F | Rosa Blanca/Cojedes | VE |
| **H58** | 1 | *An. albitarsis* F | San José del Guaviare/Guaviare | CO |
| **H59** | 1 | *An. albitarsis* F | San José del Guaviare/Guaviare | CO |
| **H60** | 1 | *An. albitarsis* F | Puerto Carreño/Vichada | CO |
| **H61** | 1 | *An. albitarsis* F | Puerto Carreño/Vichada | CO |
| **H62** | 1 | *An. albitarsis* F | San José del Guaviare/Guaviare | CO |
| **H63** | 2 | *An. albitarsis* F | San José del Guaviare/Guaviare (1), Rosa Blanca/Cojedes (1) | CO/VE |
| **H64** | 1 | *An. albitarsis* F | Jabillal/Bolívar | VE |
| **H65** | 1 | *An. albitarsis* F | San José del Guaviare/Guaviare | CO |
| **H66** | 4 | *An. albitarsis* F | Puerto Carreño/Vichada (1), Frederick Settlement/St. George (2), Jabillal/Bolívar (1) | CO/TT/VE |
| **H67** | 1 | *An. albitarsis* F | Rosa Blanca/Cojedes | VE |
| **H68** | 1 | *An. albitarsis* F | San Rafael/Bolívar | VE |
| **H69** | 1 | *An. albitarsis* F | Puerto Asís/Putumayo | CO |
| **H70** | 1 | *An. albitarsis* F | San Rafael/Bolívar | VE |
| **H71** | 2 | *An. albitarsis* F | Puerto Carreño/Vichada (1), Río Socuavo/Zulia (1) | CO/VE |
| **H72** | 1 | *An. albitarsis* F | Tibú/Norte de Santander | CO |
| **H73** | 1 | *An. albitarsis* F | Calabozo/Guárico | VE |
| **H74** | 4 | *An. albitarsis* I | El Bagre/Antioquia (3), Nechi/Antioquia (1) | CO |
| **H75** | 1 | *An. albitarsis* I | El Bagre/Antioquia | CO |
| **H76** | 2 | *An. albitarsis* I | El Bagre/Antioquia (1), Puerto Libertador/Córdoba (1) | CO |
| **H77** | 1 | *An. albitarsis* I | Zaragoza/Antioquia | CO |
| **H78** | 1 | *An. albitarsis* I | Zaragoza/Antioquia | CO |
| **H79** | 17 | *An. albitarsis* I | El Bagre/Antioquia (5), Puerto Libertador/Córdoba (2), Nechi/Antioquia (5), Santa Rosa de Lima/Bolívar (5) | CO |
| **H80** | 1 | *An. albitarsis* I | El Bagre/Antioquia | CO |
| **H81** | 1 | *An. albitarsis* I | Zaragoza/Antioquia | CO |
| **H82** | 2 | *An. albitarsis* I | El Bagre/Antioquia (1), Nechi/Antioquia (1) | CO |
| **H83** | 1 | *An. albitarsis* I | El Bagre/Antioquia | CO |
| **H84** | 2 | *An. albitarsis* I | Santa Rosa de Lima/Bolívar | CO |
| **H85** | 1 | *An. albitarsis* I | El Bagre/Antioquia | CO |
| **H86** | 1 | *An. albitarsis* I | El Bagre/Antioquia | CO |
| **H87** | 1 | *An. albitarsis* I | El Bagre/Antioquia | CO |
| **H88** | 1 | *An. albitarsis* I | El Bagre/Antioquia | CO |

Continue

| **H** | **Nº** | **Species** | **Locality/Department or State** | **Country** |
| --- | --- | --- | --- | --- |
| **H89** | 1 | *An. albitarsis* I | El Bagre/Antioquia | CO |
| **H90** | 1 | *An. albitarsis* I | El Bagre/Antioquia | CO |
| **H91** | 1 | *An. albitarsis* I | El Bagre/Antioquia | CO |
| **H92** | 1 | *An. albitarsis* I | Puerto Libertador/Córdoba | CO |
| **H93** | 1 | *An. albitarsis* I | Nechi/Antioquia | CO |
| **H94** | 1 | *An. albitarsis* I | Nechi/Antioquia | CO |
| **H95** | 1 | *An. albitarsis* I | Moñitos/Córdoba | CO |
| **H96** | 1 | *An. albitarsis* I | Puerto Libertador/Córdoba | CO |
| **H97** | 1 | *An. albitarsis* I | Puerto Libertador/Córdoba | CO |
| **H98** | 1 | *An. albitarsis* I | Puerto Libertador/Córdoba | CO |
| **H99** | 3 | *An. albitarsis* I | Río Socuavo/Zulia (1), Tibú/Norte de Santander (2) | VE/CO |
| **H100** | 3 | *An. janconnae* | MacapáAmapá (1), Ecuador/Roraima (1), Santarem/Pará (1) | BR |
| **H101** | 2 | *An. janconnae* | Boa Vista/Roraima (1), Petronila/Roraima (1) | BR |
| **H102** | 2 | *An. marajoara* | Matupá/Mato Grosso (1), Uniao de Minas/Minas Gerais (1) | BR |
| **H103** | 1 | *An. marajoara* | Peixoto de Azevedo/Mato Grosso | BR |
| **H104** | 6 | *An. marajoara* | Costa Marques/Rondonia (1), Ilha de Marajó/Pará (1), Goianesia/Pará (1), Capanema/Pará (1), Marabá/Pará (1), Macapá/Amapá (1) | BR |
| **H105** | 1 | *An. marajoara* | Jacunda/Pará | BR |
| **H106** | 1 | *An. marajoara* | Santarem/Pará | BR |

**H:** Haplotypes; **N°:** Absolute frequency of individuals observed in each haplotype. Within parentheses are the numbers of individuals observed for each haplotype in each locality. **BR:** Brazil, **CO:** Colombia, **TT:** Trinidad, **VE:** Venezuela.
