# Supplementary material for: Updating the bionomy and geographical distribution of Anopheles (Nyssorhynchus) albitarsis F: A vector of malaria parasites in northern South America
Source: PLoS One. 2021 Jun 17;16(6):e0253230. doi: 10.1371/journal.pone.0253230 (PMC8211218; doi:10.1371/journal.pone.0253230)
Supplement: S4 Table — H: Haplotypes; N°: Absolute frequency of individuals observed in each haplotype. Within parentheses are the numbers of individuals observed for each haplotype in each locality. BR: Brazil, CO: Colombia, TT: Trinidad, VE: Venezuela. (DOCX) [file pone.0253230.s004.docx]

**S4 Table.** Information of 75 haplotypes generated with the 100 DNA *mitochondrially encoded cytochrome c oxidase I* (*MT-CO1*) gene sequences database.

| **H** | **Nº** | **Species** | **Locality/Department or State** | **Country** |
| --- | --- | --- | --- | --- |
| **H1** | 1 | *An. albitarsis* F | Puerto Carreño/Vichada | CO |
| **H2** | 1 | *An. albitarsis* F | Puerto Carreño/Vichada | CO |
| **H3** | 1 | *An. albitarsis* F | San José del Guaviare/Guaviare | CO |
| **H4** | 1 | *An. albitarsis* F | Puerto Gaitán/Meta | CO |
| **H5** | 1 | *An. albitarsis* F | Rosa Blanca/Cojedes | VE |
| **H6** | 1 | *An. albitarsis* F | Río Socuavo/Zulia | VE |
| **H7** | 1 | *An. albitarsis* F | Puerto Carreño/Vichada | CO |
| **H8** | 1 | *An. albitarsis* F | Puerto Gaitán/Meta | CO |
| **H9** | 1 | *An. albitarsis* F | St. David/St. Andrew | TT |
| **H10** | 1 | *An. albitarsis* F | San José del Guaviare/Guaviare | CO |
| **H11** | 1 | *An. albitarsis* F | Jabillal/Bolívar | VE |
| **H12** | 1 | *An. albitarsis* F | Puerto Gaitán/Meta | CO |
| **H13** | 1 | *An. albitarsis* F | Tibú/Norte de Santander | CO |
| **H14** | 1 | *An. albitarsis* F | Río Socuavo/Zulia | VE |
| **H15** | 1 | *An. albitarsis* F | Puerto Asís/Putumayo | CO |
| **H16** | 1 | *An. albitarsis* F | Puerto Carreño/Vichada | CO |
| **H17** | 1 | *An. albitarsis* F | Tibú/Norte de Santander | CO |
| **H18** | 1 | *An. albitarsis* F | Tibú/Norte de Santander | CO |
| **H19** | 1 | *An. albitarsis* F | Río Boconó/Portuguesa | VE |
| **H20** | 1 | *An. albitarsis* F | Tibú/Norte de Santander | CO |
| **H21** | 1 | *An. albitarsis* F | Puerto Carreño/Vichada | CO |
| **H22** | 1 | *An. albitarsis* F | Rosa Blanca/Cojedes | VE |
| **H23** | 2 | *An. albitarsis* F | Frederick Settlement/St. George (2) | TT |
| **H24** | 1 | *An. albitarsis* F | San Rafael/Bolívar | VE |
| **H25** | 1 | *An. albitarsis* F | Puerto Asís/Putumayo | CO |
| **H26** | 1 | *An. albitarsis* F | San Rafael/Bolívar | VE |
| **H27** | 2 | *An. albitarsis* F | San José del Guaviare/Guaviare (1); Rosa Blanca/Cojedes (1) | CO/VE |
| **H28** | 1 | *An. albitarsis* F | Tibú/Norte de Santander | CO |
| **H29** | 1 | *An. albitarsis* F | Calabozo/Guárico | VE |
| **H30** | 1 | *An. albitarsis* F | Río Socuavo/Zulia | VE |
| **H31** | 1 | *An. albitarsis* F | Jabillal/Bolívar | VE |
| **H32** | 1 | *An. albitarsis* F | Tibú/Norte de Santander | CO |
| **H33** | 1 | *An. albitarsis* F | Jabillal/Bolívar | VE |
| **H34** | 1 | *An. albitarsis* F | Cruce Granada/Meta | CO |
| **H35** | 5 | *An. albitarsis* I | Nechi/Antioquia (2), Santa Rosa de Lima/Bolívar (2), Puerto Libertador/Córdoba (1) | CO |
| **H36** | 1 | *An. albitarsis* I | Zaragoza/Antioquia | CO |
| **H37** | 1 | *An. albitarsis* I | El Bagre/Antioquia | CO |
| **H38** | 1 | *An. albitarsis* I | El Bagre/Antioquia | CO |
| **H39** | 1 | *An. albitarsis* I | Zaragoza/Antioquia | CO |
| **H40** | 1 | *An. albitarsis* I | Nechi/Antioquia | CO |
| **H41** | 1 | *An. albitarsis* I | Moñitos/Córdoba | CO |
| **H42** | 3 | *An. albitarsis* I | Río Socuavo/Zulia (1); Tibú/Norte de Santander (2) | VE/CO |
| **H43** | 3 | *An. janconnae* | Ecuador/Roraima (1), Santarem/Pará (1), Macapá/Amapá (1) | BR |

Continue

| **H** | **Nº** | **Species** | **Locality/Department or State** | **Country** |
| --- | --- | --- | --- | --- |
| **H44** | 2 | *An. janconnae* | Boa Vista/Roraima (1), Petronila/Roraima (1) | BR |
| **H45** | 3 | *An. albitarsis* G | Itaquara/Bahia (1), Manaus/Amazonas (1), Río Paru/Pará (1) | BR |
| **H46** | 1 | *An. albitarsis* G | Campina/Pará (1) | BR |
| **H47** | 3 | *An. albitarsis* G | Itaituba/Pará (1), Monte Alegre/Pará (1), Parintins/Amazonas (1) | BR |
| **H48** | 1 | *An. albitarsis* G | Uruara/Pará | BR |
| **H49** | 1 | *An. albitarsis* H | Peixoto de Azevedo/Mato Grosso | BR |
| **H50** | 1 | *An. albitarsis* H | Lagoa da Confusao/Tocantins | BR |
| **H51** | 1 | *An. albitarsis* H | Balsas/Maranhao | BR |
| **H52** | 1 | *An. albitarsis* H | Costa Marques/Rondonia | BR |
| **H53** | 1 | *An. albitarsis* H | Marabá/Pará | BR |
| **H54** | 2 | *An. marajoara* | Matupá/Mato Grosso (1), Uniao de Minas/Minas Gerais (1) | BR |
| **H55** | 1 | *An. marajoara* | Peixoto de Azevedo/Mato Grosso | BR |
| **H56** | 6 | *An. marajoara* | Costa Marques/Rondonia (1), Ilha de Marajó/Pará (1), Goianesia/Pará (1), Capanema/Pará (1), Marabá/Pará (1), Macapá/Amapá (1) | BR |
| **H57** | 1 | *An. marajoara* | Jacunda/Pará | BR |
| **H58** | 1 | *An. marajoara* | Santarem/Pará | BR |
| **H59** | 1 | *An. deaneorum* | Acrelândia/Acre | BR |
| **H60** | 1 | *An. deaneorum* | Ariquemes/Rondonia | BR |
| **H61** | 1 | *An. deaneorum* | Nr Guaira/Paraná | BR |
| **H62** | 1 | *An. deaneorum* | Laguna Brava/Corrientes | AR |
| **H63** | 1 | *An. deaneorum* | Aquidauana/Mato Grosso do Sul | BR |
| **H64** | 1 | *An. albitarsis* s.s. | Macaranduba/Santa Catarina | BR |
| **H65** | 1 | *An. albitarsis* s.s. | Mercedes/Corrientes | AR |
| **H66** | 1 | *An. albitarsis* s.s. | Río Acaray/Alto Paraná | PY |
| **H67** | 1 | *An. albitarsis* s.s. | Baradero/Buenos Aires | AR |
| **H68** | 1 | *An. albitarsis* s.s. | Santa Helena/Paraná | BR |
| **H69** | 2 | *An. albitarsis* s.s. | Ilha Comprida/Sao Paulo (1), Santa Luzia/Bahia (1) | BR |
| **H70** | 1 | *An. oryzalimnetes* | Dourado/Sao Paulo | BR |
| **H71** | 1 | *An. oryzalimnetes* | Jaguaré/Espirito Santo | BR |
| **H72** | 2 | *An. oryzalimnetes* | Morro da Panela/Río de Janeiro (1), Foz do Iguaçú, Paraná (1) | BR |
| **H73** | 1 | *An. oryzalimnetes* | Paraipaba/Ceará | BR |
| **H74** | 2 | *An. oryzalimnetes* | Primavera/Pará (1), Peixoto de Acevedo/Mato Grosso (1) | BR |
| **H75** | 1 | *An. oryzalimnetes* | Luque/Alto Paraná | BR |

**H:** Haplotypes; **N°:** Absolute frequency of individuals observed in each haplotype. Within parentheses are the numbers of individuals observed for each haplotype in each locality. **AR:** Argentina, **BR:** Brazil, **CO:** Colombia, **PY:** Paraguay, **TT:** Trinidad, **VE:** Venezuela.
