# Supplementary figures and images for: Updating the bionomy and geographical distribution of Anopheles (Nyssorhynchus) albitarsis F: A vector of malaria parasites in northern South America
Source: PLoS One. 2021 Jun 17;16(6):e0253230. doi: 10.1371/journal.pone.0253230 (PMC8211218; doi:10.1371/journal.pone.0253230)

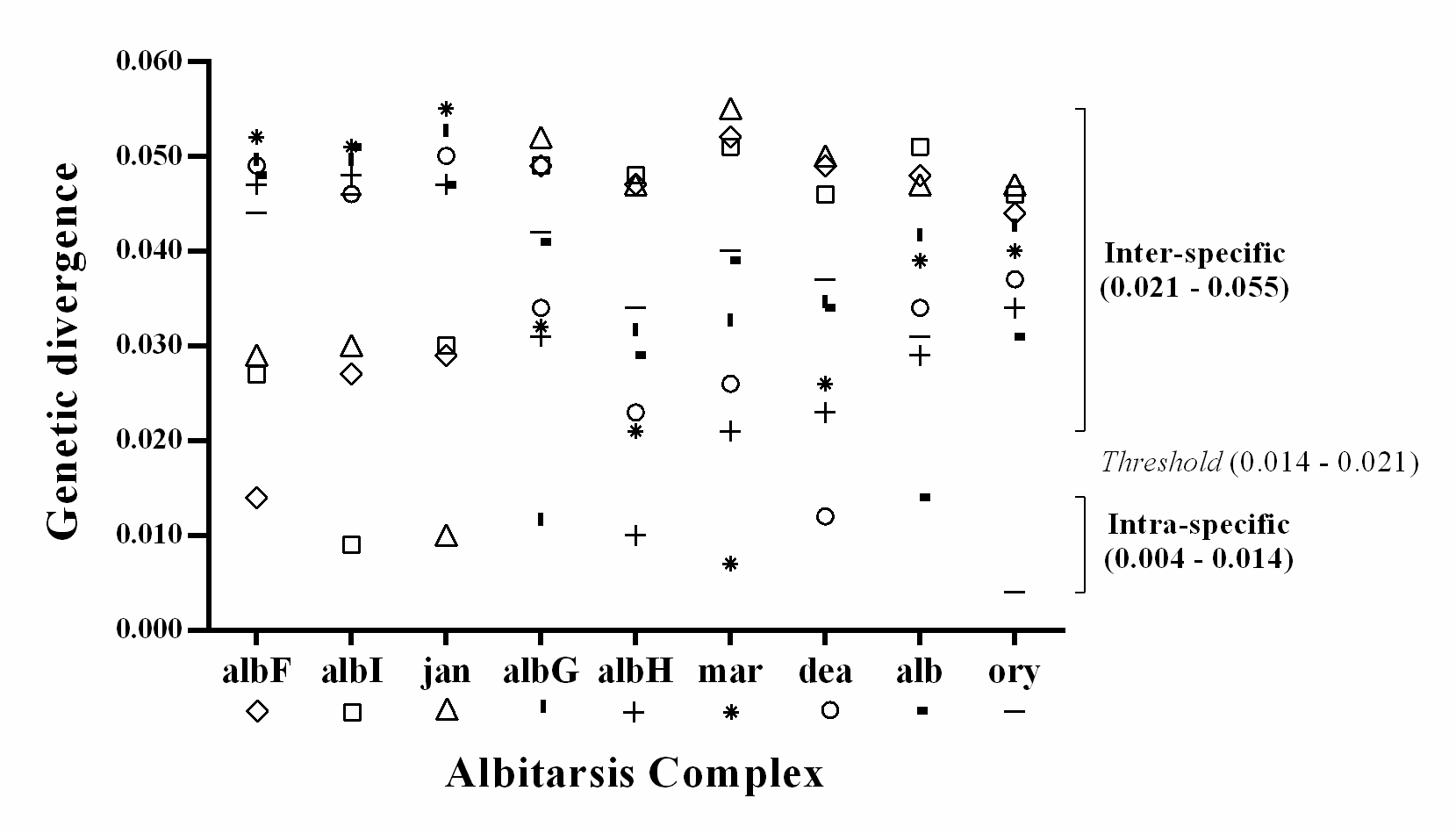

Supplement: S1 Fig — Barcode gap analysis of all species within the Anopheles albitarsis complex, plots are based in distance matrices of the clusters determined using NJ-K2P distances. Y-axis: genetic divergence and X-axis clusters. alb: An. albitarsis s.s.; ory: An. oryzalimnetes; mar: An. marajoara; dea: An. deaneorum; jan: An. janconnae; albF: An. albitarsis F; albG: An. albitarsis G; albH: An. albitarsis H; albI: An. albitarsis I. (TIF) [file pone.0253230.s005.tif]
